# Additive Function of *Vibrio vulnificus* MARTX_Vv_ and VvhA Cytolysins Promotes Rapid Growth and Epithelial Tissue Necrosis During Intestinal Infection

**DOI:** 10.1371/journal.ppat.1002581

**Published:** 2012-03-22

**Authors:** Hee-Gon Jeong, Karla J. F. Satchell

**Affiliations:** Department of Microbiology-Immunology, Feinberg School of Medicine, Northwestern University, Chicago, Illinois, United States of America; University of Illinois, United States of America

## Abstract

*Vibrio vulnificus* is a pathogen that causes both severe necrotizing wound infections and life-threatening food-borne infections. Food-borne infection is particularly lethal as the infection can progress rapidly to primary septicemia resulting in death from septic shock and multiorgan failure. In this study, we use both bioluminescence whole animal imaging and *V. vulnificus* bacterial colonization of orally infected mice to demonstrate that the secreted multifunctional-autoprocessing RTX toxin (MARTX_Vv_) and the cytolysin/hemolysin VvhA of clinical isolate CMCP6 have an important function in the gut to promote early *in vivo* growth and dissemination of this pathogen from the small intestine to other organs. Using histopathology, we find that both cytotoxins can cause villi disruption, epithelial necrosis, and inflammation in the mouse small intestine. A double mutant deleted of genes for both cytotoxins was essentially avirulent, did not cause intestinal epithelial tissue damage, and was cleared from infected mice by 36 hours by an effective immune response. Therefore, MARTX_Vv_ and VvhA seem to play an additive role for pathogenesis of CMCP6 causing intestinal tissue damage and inflammation that then promotes dissemination of the infecting bacteria to the bloodstream and other organs. In the absence of these two secreted factors, we propose that this bacterium is unable to cause intestinal infection in humans.

## Author Summary

*Vibrio vulnificus* causes disease both by infection of wounds from seawater and by consumption of contaminated foods, especially oysters. Wound infection results in necrotizing fasciitis and edema in extremities with mortality of ∼25% as the incidence of septicemia is low. Contaminated food consumption by contrast can lead to highly invasive infections that progress rapidly from an intestinal infection to primary septicemia. Case-fatality rates are ≥50%, with rates as high as 100% in individuals who receive no antibiotic therapy. The aim of this study is to elucidate virulence mechanisms of food-borne infection of the most highly virulent strains of *V. vulnificus*. We developed a novel intragastric infection model for a highly virulent clinical isolate from Korea in which we can observe the bacterial load in live mice and applied this to study of wild type and strains genetically altered to delete genes for two secreted cytotoxins. Using this model, we show that both the multifunctional-autoprocessing RTX toxin (MARTX_Vv_) and the cytolysin VvhA contribute to rapid *in vivo* growth of bacteria and that the presence of these factors directly correlates with mouse mortality. These exotoxins are then directly linked to intestinal damage and inflammation.

## Introduction

*Vibrio vulnificus* is a motile, Gram-negative, opportunistic human pathogen capable of causing severe to life-threatening infection in individuals with predisposing conditions, including liver damage, hereditary hemochromatosis and compromised immune systems [1]–[3]. Infection can result from consumption of contaminated seafood or from exposing an open wound to water harboring the pathogen. Wound infection can progress to edema, cellulitis, ecchymoses and necrotizing fasciitis at the site of infection [4], [5]. The mortality of wound infection is about 25% because primary septicemia does not frequently occur [5], [6]. By contrast, *V. vulnificus* food-borne infection rapidly progresses to primary septicemia with symptoms that include high fever, chills, decreased blood pressure and septic shock [5], [7]–[9]. These infections result in a much higher mortality rate (≥50%) with rates as high as 100% in the absence of antibiotic therapy [5], [6], [10]. Hence, a critical aspect of *V. vulnificus* pathogenesis is its ability to infect a host via the gastrointestinal tract and then rapidly spread from the small intestine to the blood stream.

Although several secreted virulence factors of *V. vulnificus* have been identified [3], [11], only two have been previously associated with increased death during intestinal infection: the secreted cytolytic/hemolysin pore-forming toxin encoded by *vvhA* [12] and the multifunctional autoprocessing RTX (MARTX_Vv_) toxin encoded by gene *rtxA1* [13]–[15]. *In vitro*, both of these toxins are cytolysins associated with lysis of a variety of cell types including erythrocytes, epithelial cells and macrophages, albeit by different molecular mechanisms [12], [16]–[22].

The role of these toxins *in vivo* during infection has been less well-characterized. When injected directly to the bloodstream, purified VvhA is lethal at sub-µg levels and causes hypotension and tachycardia, along with skin and pulmonary damage [12], [23]. However, deletion of *vvhA* from *V. vulnificus* had either a slight or no defect in virulence when delivered intraperitoneally (i.p.) and no defect when delivered intradermally (i.d.) [23]. When delivered intragastically (i.g.) to neutropenic mice, loss of *vvhA* resulted in a detectable, albeit modest, 4–5 fold increase in median lethal dose (LD_50_) [12], [23].

In comparison to VvhA, MARTX_Vv_ has been shown to have a significantly greater contribution to mouse lethality. A mutant in *rtxA1* has a 100- to 500-fold increase in LD_50_ compared to wild-type when inoculated i.p. [14], [17], [18] and a 13-fold increase when inoculated subcutaneously (s.c.) [19]. A deletion of *rtxA1* caused a 180 to 2600-fold increase in LD_50_ in an i.g. infection model with the contribution of the gene deletion to virulence varying depending on the specific toxin variant that is expressed [15]. Comparison across different studies suggest that the MARTX_Vv_ toxin is the most significant virulence factor of *V. vulnificus* and both MARTX_Vv_ and VvhA exert a greater effect on i.g. and septicemic infection compared to i.p., s.c. or i.d. infection.

In this study, we sought to understand how cytotoxins MARTX_Vv_ and VvhA contribute to food-borne infection by highly virulent *V. vulnificus* strains that produce a particularly potent variant of the MARTX_Vv_ toxin [15]. We used bioluminescence imaging (BLI) and measurement of bacterial colonization to monitor early events in growth and dissemination of *V. vulnificus* strain CMCP6 in mice after i.g. infection. This study shows that both MARTX_Vv_ and VvhA from CMCP6 contribute to the onset of colonization and to significant bacterial growth sooner after inoculation. These data are consistent with a role of both toxins in disabling innate immune cells in the small intestine allowing for more rapid growth. However, the effect of the toxins is not limited to innate cells as these toxins are also here shown to directly cause epithelial tissue damage. The combination of rapid growth and tissue damage is essential for the dissemination to the bloodstream earlier during the infection cycle and this rapid dissemination is the leading factor promoting death.

## Results

### Characterization of luciferase-expressing *V. vulnificus*

*V. vulnificus* CMCP6 can cause lethal infection of adult mice inoculated i.g. [15]. To more directly measure how disease progresses during early infection, we transformed *V. vulnificus* CMCP6 with plasmid pHGJ1 that expresses the *Photorhabdus luminescens lux* genes from the constitutively active *Vibrio cholerae ompC* promoter (see material and methods). The LD_50_ for the resulting strain CMCP6*lux* (HG0905) was 3.1×10^5^ CFU (Table S1), about 13-fold higher than the LD_50_ of 2.4×10^4^ CFU previously determined for the parent strain CMCP6 in this mouse infection model [15]. The presence of the plasmid also caused an *in vitro* defect in growth in antibiotic-free culture media (Figure S1). The difference in both *in vitro* growth and *in vivo* virulence between the parent strain CMCP6 and CMCP6*lux* (HG0905) is likely due to spontaneous bacterial death upon plasmid loss. To maintain the *lux* reporter without antibiotic selection, the *lux* plasmid (pHGJ1) carries the *hok/sok* plasmid addiction system [24]. Bacteria that lose the plasmid during cell division will die upon dilution of the less stable antitoxin. The advantage of this system is that only bacteria that produce luciferase survive and thus there is no contribution to infection from bacteria that are not *lux* ^+^. In addition, the expression of the luciferase genes also probably contribute to the reduced virulence of HG0905 compared to CMCP6 since a CMCP6 that carries a plasmid deleted of the *lux* genes (HG0909) was slightly more virulent than HG0905 (Figure S1).

**Figure 1:**
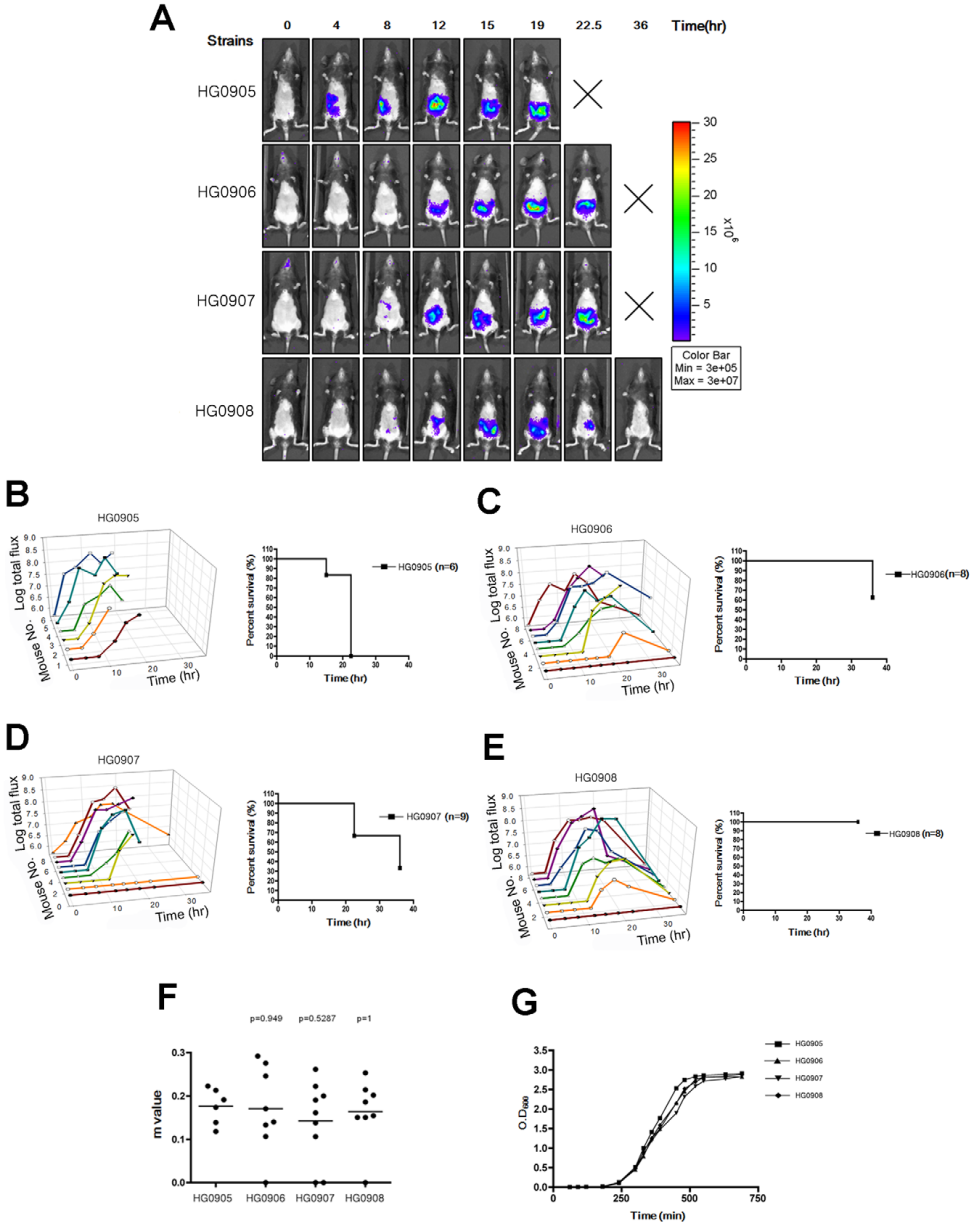
Bioluminescence quantification and lethality measurement in mice infected with *V. vulnificus* strains containing pHGJ1. (A) 5–6 weeks old C57BL/6 mice were infected with 1×10^6^ CFU of HG0905 (CMCP6*lux*) and isogenic mutants HG0906 (*ΔrtxA1*), HG0907 (*ΔvvhBA*) or HG0908 (*ΔrtxA1vvhBA*) strains i.g. as indicated. Representative images were acquired at 0, 4, 8, 12, 15, 19, 22.5 and 36 hr post infection, and the color scale of total flux represents the photons s^−1^ cm^−2^ sr^−1^ for combined images is shown. (B–E) Survival curve and light emission from every individual mouse infected with HG0905 (B), HG0906 (C), HG0907 (D) and HG0908 (E) were measured. (F) Slopes (m) between onset and peak point from total flux of individual mice were measured. (G) *In vitro* growth curves of strains in LB broth at 30°C with shaking of HG0905, HG0906, HG0907 and HG0908 for OD_600_.

In this paper, we compared *lux*+ strains derived from parent strain CMCP6 and we confirmed that there was no *in vitro* growth difference in mutant strains CMCP6*luxΔrtxA1* (HG0906), CMCP6*luxΔvvhBA* (HG0907) and CMCP6*luxΔrtxA1vvhBA* (HG0908) containing plasmid pHGJ1 in antibiotic-free culture media (Figure 1G) compared to the isogenic parent CMCP*lux* (HG0905). Further, we confirmed by lactate dehydrogenase (LDH) release assays that the mutants carrying pHGJ1 have defects in HeLa cell lysis consistent with previous findings [14], [18]. Specifically, the Δ*rtxA1* mutation reduced rapid HeLa cell lysis while the Δ*vvhBA* mutation eliminated slow cell lysis. The double mutant did not lyse cells (Figure S2).

The effect on virulence of the *lux*+ plasmids was less evident in the mutant strains since these strains likely are not growing *in vivo* and thus less likely to lose the plasmid during rapid replication. Thus, the LD_50_ for CMCP6*lux*Δ*rtxA1* (HG0906) matched our previously determined LD_50_ of 8.0×10^7^ CFU for plasmid-free CMCP6Δ*rtxA1* and thus the deletion of *rtxA1* exhibited only a 260-fold effect on virulence due to the reduced virulence of the isogenic wild type. Deletion of *vvhA* caused a 61-fold decrease in virulence with an LD_50_ of 1.9×10^7^. This result was surprising since previous studies of i.g. infection with a Δ*vvhA* mutant in isolate YJ016 revealed only a modest 5-fold virulence defect [12]. However, the previous study was conducted in highly susceptible iron-overloaded, neutropenic mice, which may have masked the importance of this factor for intestinal infection. Consistent with a role of both factors in infection by CMCP6, the CMCP6*lux*Δ*rtxA1*Δ*vvhBA* double mutant (HG0908) was essentially avirulent with an LD_50_>10^9^ (Table S1).

### *In vivo* expansion of *V. vulnificus* after i.g. infection

To monitor how rapidly *V. vulnificus* bacteria expand *in vivo*, bioluminescence from mice infected with CMCP6*lux* (HG0905) was observed and quantified at defined intervals using an IVIS 100 bioluminescence imager (Xenogen Corp.). As previously described by others [25], use of anesthesia during orogastric inoculation can result in accidental lung infection due to contamination of the larynx during infection. In pilot studies, we similarly found that some mice developed lung infection. These mice usually died rapidly, often by 6 hours after infection. In our study, 3 mice infected with HG0905 developed infection of the lung detectable by IVIS imaging by 4 hr. These mice were euthanized and removed from analysis. All other mice did not show detectable lung infection by IVIS imaging.

At a dose of 1×10^6^ CFU, 100% of mice with an intestinal infection died between 12 and 22.5 hr post-inoculation (Figure 1B). Prior to death, all of the CMCP6*lux* (HG0905) inoculated mice (6/6) showed detectable levels of photon flux. Two of the mice reached our preset detection limit by 4 hr, 3 mice by 8 hr, and all mice had detectable levels by 12 hr (Figure 1A and B). Of note, all mice showed a steady rise (mean slope between onset and peak (m) equals 0.174; Figure 1F) in light emission until the animal was sacrificed for severe morbidity between 12 and 22.5 hr demonstrating constant replication of the wild-type bacteria. However, even though all mice reached at least 7.3 RLU (Relative Luminescence Unit on a logarithmic scale; Luminescence Unit represents the photons s^−1^ cm^−2^ sr^−1^) before death, attainment of this level did not predict eminent death as three mice survived for 4–22.5 hr after crossing this threshold.

**Figure 2:**
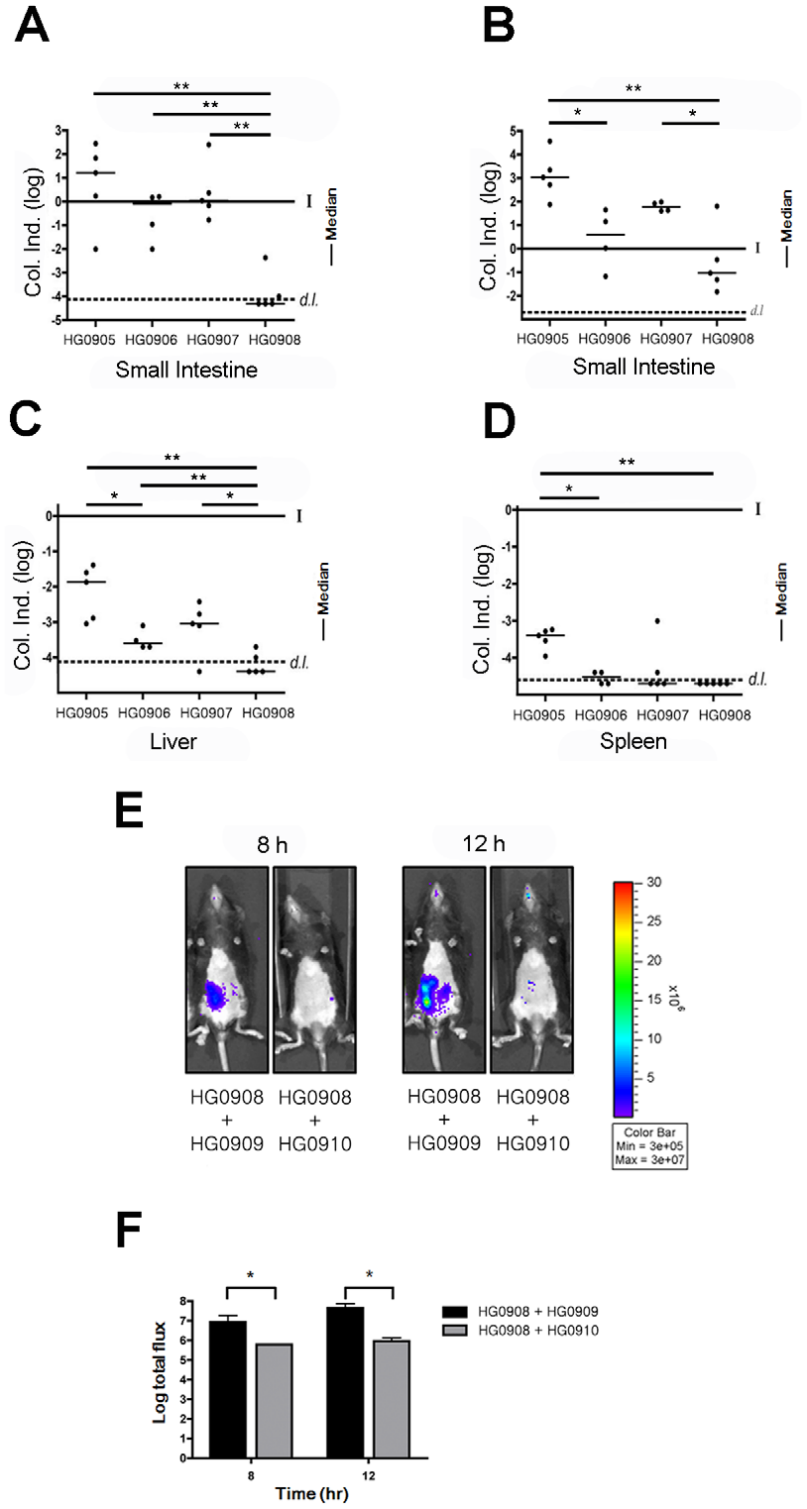
Dynamics of bacterial colonization and dissemination. C57BL/6 mice (5–6 weeks old) were infected with 1×10^6^ CFU of HG0905 (CMCP6*lux*) and isogenic mutants HG0906 (*ΔrtxA1*), HG0907 (*ΔvvhBA*) and HG0908 (*ΔrtxA1vvhBA*). Small intestines after 8 hr (A) and 12 hr (B) post infection, liver (C) and spleen (D) were collected, homogenized and plated for CFU counting (*, *p*<0.05; **, *p*<0.01). Values are reported as a log colonization index (Col. Ind.), which is defined as the log of the number of recovered CFU divided by the number of input CFU. Solid line at 0 indicates that CFU recovered was identical to the input CFU (I) while values above the line signify growth and values below the line indicate clearance. Values below the dashed line (*d.l.*) were below the detection limit. (E) Co-infections experiments were performed by inoculation of a mouse with the bacterial suspension prepared by mixing equal numbers of the HG0908 (*lux*+ *ΔrtxA1vvhBA*) and HG0909 (*lux* ^−^ CMCP6), and also HG0908 and HG0910 (*lux* ^−^ *ΔrtxA1vvhBA*). BLI from mice were acquired after 8 hr and 12 hr. (F) Median values of total flux were also quantified.

To demonstrate that photon flux is representative of changes in intestinal colonization, in a separate experiment, 5 mice inoculated with 10^6^ CFU were euthanized at both 8 and 12 hr. In accordance with flux readout in the previous experiment (Figure 1A and B), there was variability in recovered CFU from the small intestine at 8 hr ranging from 10^4^–10^8^ CFU or −2 to +3 log unit change from the inoculation dose (Figure 2A). By 12 hr, all mice were colonized above the infection dose representing 2–5 log units growth (Figure 2B). In addition, there was dissemination of the bacteria to the liver and spleen by 8 hr, indicating progression to septicemia in all mice during the earliest stages of infection (Figure 2C and D). Overall, *V. vulnificus* CMCP6*lux* (HG0905) was shown to expand *in vivo* and this rapid growth occurred coincident with dissemination to other tissues shortly after inoculation.

### Loss of MARTX_Vv_ results in delayed growth and reduced virulence

To determine if MARTX_Vv_ is the factor that promotes the rapid growth seen in mice after i.g. infection, we monitored the effect of deletion of the *rtxA1* gene from CMCP6*lux* on disease progression. The most apparent phenotype of the resulting strain HG0906 compared to CMCP6*lux* (HG0905) was a delay in the time required to the BLI detection limit (Figure 1A). One of 9 mice was sacrificed due to lung infection and one mouse was not infected. Among the 7 infected mice, light production was detectable in only one (14%) by 4 hr and 3/7 (43%) of mice by 8 hr (Figure 1C). Four mice (50%) showed delayed onset with detectable light emission only after 8–12 hr. After onset, the average rate of growth in all infected mice was similar to wild-type (m = 171, Figure 1F). However, unlike CMCP6*lux* (HG0905) infected mice, 3 of 7 mice ultimately survived to 36 hr despite the *in vivo* bacterial load. In addition, several of the mice succumbed only late in the experiment indicating, as suggested by LD_50_ studies (Table S1), that more mice might have survived except for the stress imposed by repeated anaesthetic regimen necessary for imaging. Thus, the major effect of loss of the MARTX_Vv_ toxin was delayed onset of bacterial growth to detectable levels. Further, among the mice that attained high bacterial loads, half failed to progress to death and the mice cleared the infections. As further evidence for delayed growth, there was a trend toward reduced colonization of the small intestine by the CMCP6*lux*Δ*rtxA1* mutant (HG0906) at 8 hr that reached statistical significance by 12 hr (Figure 2A and B). In addition, by 8 hr, there was significantly reduced dissemination of bacteria to the liver and spleen (Figure 2C and D). Overall, our results suggest that loss of *rtxA1* results in an inability to consistently establish an infection that can progress to other organs shortly after ingestion of bacteria and thus the infections are delayed and less severe at least 50% of the time.

### Cytolysin/hemolysin VvhA also significantly contributes to growth and dissemination *in vivo*

To test if VvhA also contributes to infection, we next tested a CMCP6*lux*Δ*vvhBA* mutant (HG0907). Results were intermediate between CMCP6*lux* and the isogenic Δ*rtxA1* mutant with 7 of 9 mice showing increased light emission beginning 4–12 hr after inoculation rising to values greater than 7.3 RLU. Similar to both CMCP6*lux* and the isogenic Δ*rtxA1* mutant, the 7 mice successfully infected with CMCP6*lux*Δ*vvhBA* mutant showed a similar rate of increasing light emission with other strains indicating it does grow *in vivo* (m = 0.142; Figure 1F). 1 of these 7 mice reversed course and began to clear the infection while the other 6 succumbed to infection by 22.5 hr. When assessed for colonization, there was a consistent trend for reduced colonization of CMCP6*lux*Δ*vvhBA* mutant (HG0907) in the intestine at 8 hr and 12 hr (Figure 2A and B) and reduced dissemination during early infection to the liver and spleen but these values did not achieve statistical significance (Figure 2C and D). Thus, mice infected with the Δ*vvhBA* mutant showed detectable decreases in numerous parameters of infection including delayed and reduced death but this cytolysin does not exert the same impact on progression of CMCP6 infection as MARTX_Vv_.

Despite its minimal effect when the *rtxA1* is intact, expression of VvhBA by CMCP6*lux* does account for the residual virulence of the CMCP6*lux*Δ*rtxA1* mutant. A CMCP6*lux* double mutant eliminating both *vvhBA* and *rtxA1* (HG0908) was nonlethal in mice at 1×10^6^ CFU (Figure 1E) except for one mouse sacrificed due to lung infection. Three of the mice were overall defective for bacterial growth and did not achieve 7.3 RLU at any time point and 1 was not infected at all. In 2/8 (25%) mice there was long 12 hour delay to detectable light production (Figure 1E). When bacteria did expand *in vivo*, the mean slope from onset of detection to peak (m = 0.164) was similar with those of wild type (Figure 1F). However, in all cases where mice did achieve high bacteria loads, the emission of light reversed from a peak between 15 and 22.5 hr post-inoculation and all mice ultimately cleared the infection.

When tested for colonization, CFU recovered from the small intestine were significantly reduced at both 8 and 12 hr (Figure 2A and B) and the bacteria did not disseminate to the liver and spleen by 8 hr (Figure 2C and D). Overall, these data indicate that in *V. vulnificus* strain CMCP6, MARTX_Vv_ in conjunction with a secondary additive contribution from VvhA is essential during the early stages of infection to promote initiation of the infection and dissemination to the bloodstream.

### In vivo growth of double cytolysin mutant HG0908 is restored by co-infection with CMCP6*lux*

Lack of bacterial growth during *in vivo* infection due to loss of secreted factors can often be restored by co-infection wherein mutant bacteria benefit from alteration to the host environment by the co-infecting strain. These data can reveal that mutant bacteria are not defective in their ability to replicate in vivo per se, but lack the capacity to modify the host environment to promote their growth. To test if HG0908 could be restored for *in vivo* growth by co-infection, we transferred a plasmid from which the *luxCDABE* operon was deleted (pHGJ2) into CMCP6 and the double cytolysin mutant strains and competed strains 1∶1 with the *lux* ^+^ double mutant (HG0908). Thereby, if HG0908 is rescued by co-infection, total flux during co-infection should increase since all light signal would originate from HG0908 and not the cytolysin producing co-infecting strain.

When 5×10^5^ CFU of *lux^−^* wild-type (HG0909) and 5×10^5^ CFU of *lux* ^+^ double mutant (HG0908) were co-inoculated, median light production produced by the double mutant was 6.9 RLU at 8 hr post-infection and reached 7.6 RLU after 12 hr infection. By contrast, mice infected with 5×10^5^ CFU of *lux*+ double mutant (HG0908) and 5×10^5^ CFU *lux^−^* double mutant (HG0910) was 5.7 RLU at 8 hr post-infection and 5.9 RLU after 12 hr (Figure 2E and F).

Thus, the *in vivo* growth defect of the double cytolysin mutant HG0908 can be restored by co-infection with a cytolysin producing strain indicating that HG0908 is not defective in its ability to replicate *in vivo* but in its ability to modify the host environment.

### Histopathological evaluation of ileum tissue reveals MARTX_Vv_ and VvhA cause epithelial cell damage

**Figure 3:**
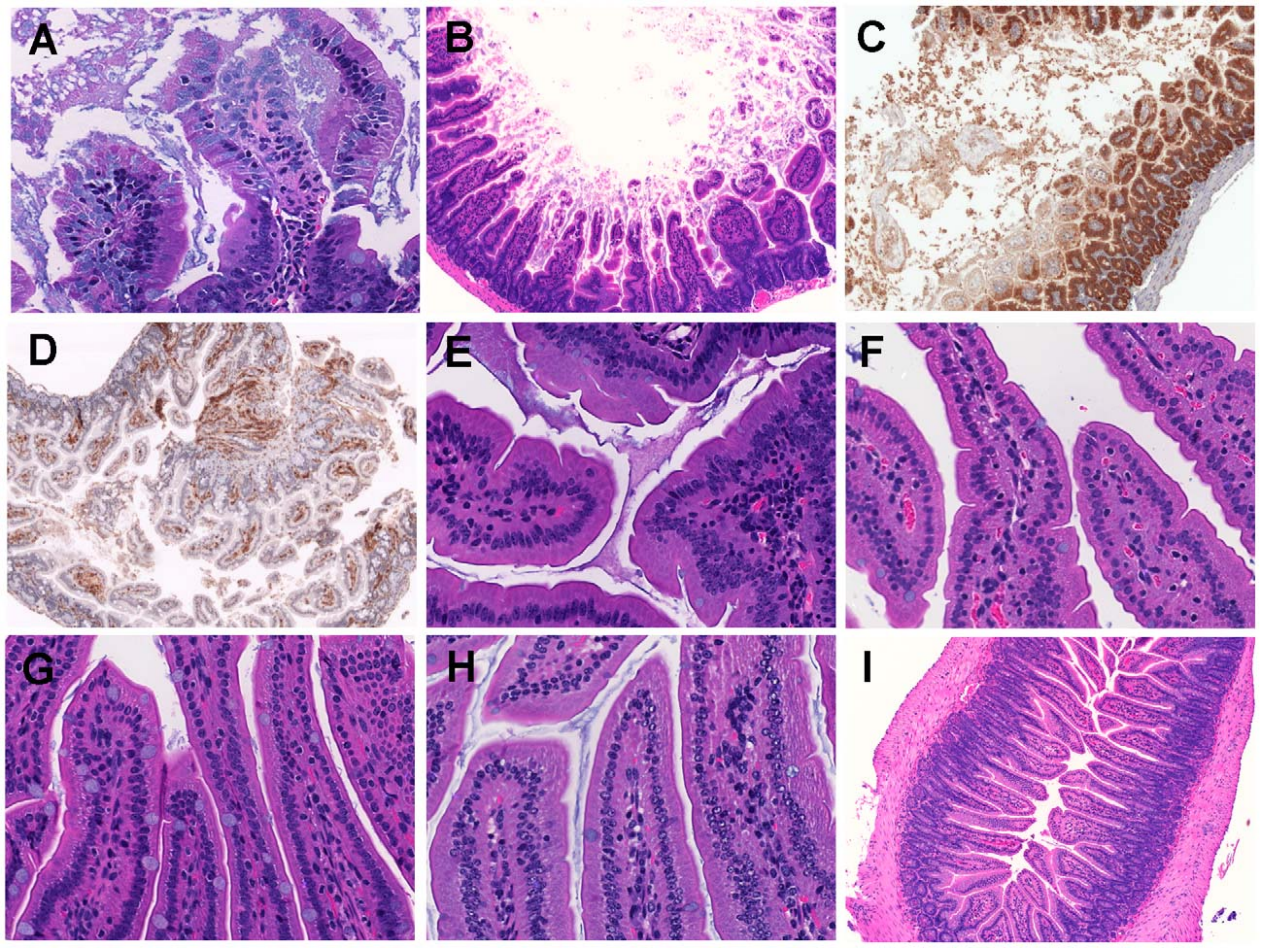
Histopathological examination of mice small intestine inoculated with *V. vulnificus* strains. Micrographs of ileum of mice inoculated i.g. with 1×10^6^ CFU of HG0905 (CMCP6*lux*; A to D) and isogenic mutants HG0906 (*ΔrtxA1*; E), HG0907 (*ΔvvhBA*; F), or HG0908 (*ΔrtxA1vvhBA*; G). Mice ileum infected with PBS were used for the negative control (H and I). (A) Infiltration of lamina propria in mice ileum infected with HG0905. Sloughed villi, necrotic debris epithelial cells and leukocyte are abundant in lumen, which are stained by H&E (B), anti-ß-catenin antibody (brown) (C) and anti-CD45 antibody (brown) (D). Both magnified views of villi infected with HG0906 (E) and HG0907 (F) showed a little swelling. (G) Intact villi were observed from mice infected with HG0908 (magnification of A, E, F, G and H, 400×; magnification of B, C, D and I, 200×).

**Figure 4:**
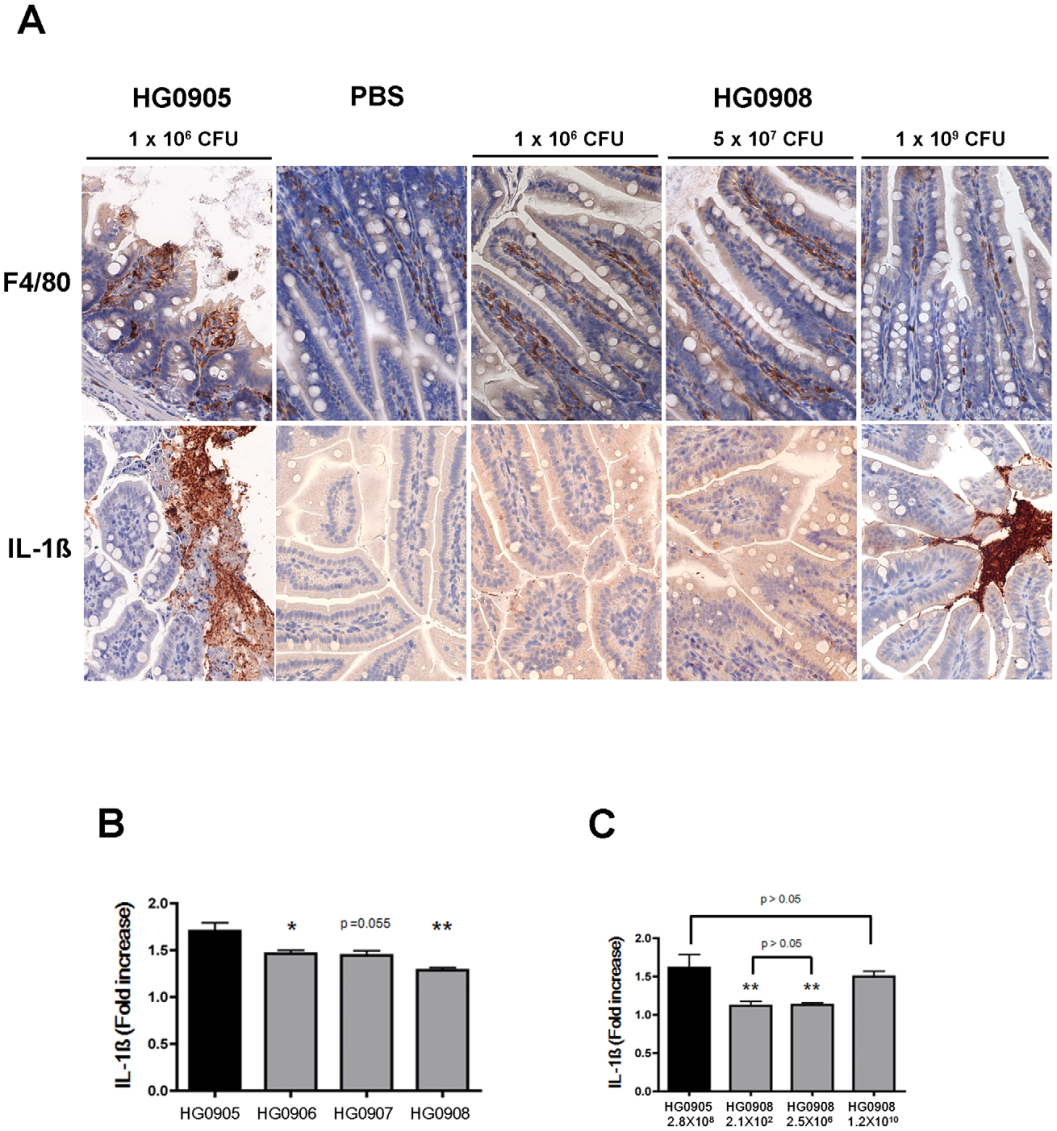
Comparison of HG0905 effect on villi, macrophage and IL-1ß expression to HG0908. (A) Immunohistochemistry staining of ileum (magnification 400×) for F4/80 antibody (brown) and IL-1ß antibody (brown) in mice infected with 1×10^6^ CFU of HG0905 (CMCP6*lux*) or various doses of HG0908. (B and C) Fold increase of IL-1ß secretion over PBS control in small intestine from mice infected with (B) 1×10^6^ CFU of indicated strain or (C) varying concentrations of HG0905 or HG0908 as indicated were measured by ELISA after 8 hr infection (*, *p*<0.05; **, *p*<0.01).

We have demonstrated that MARTX_Vv_ and VvhA of *V. vulnificus* CMCP6 are required for earlier onset of *in vivo* growth after i.g. inoculation. This result is consistent with a recent study conducted by s.c. infection to model wound-induced infection using strain YJ016. The s.c. study of YJ016 suggested the requirement for MARTX_Vv_ during infection is primarily for protection from phagocytes to promote growth [19]. This conclusion seems to conflict with evidence that cytotoxins of CMCP6, YJ016, and other *V. vulnificus* strains are linked to lysis of both epithelial cells and macrophages *in vitro* [14], [16]–[19], [21]. To reveal whether the cytotoxins have an additional role beyond promoting rapid growth during intestinal infection, mice were inoculated with a lethal dose of CMCP6*lux* (HG0905) and the terminal ileal tissue was collected after 8 hr infection for various histopathological staining. Severe disruption of the intestinal barrier occurred in the ileum infected with HG0905 (Figure 3A–C) with many broken villi and barrier disruptions, consistent with pathology reported in earlier studies in neutropenic mice using strain YJ016 [12]. Excessive amounts of epithelial cell debris and heavy cellular infiltration of lamina propria were observed in the lumen and mucosa of the ileum from mice infected with the wild type (Figure 3A–C). Staining with anti-CD45 showed extensive influx of monocytes and other immune cells to the tissue and the lumen (Figure 3D). Within the destroyed tissue, F4/80 positive macrophages are present and proinflammatory cytokine IL-1β is secreted and found distributed in the ruptured tissue (Figure 4A). The lumen is filled with epithelial debris (stained positive with β-catenin), lysed macrophages, and IL-1β presumably release from necrotic macrophages (Figure 3C and 4A).

By contrast, mice infected with 1×10^6^ CFU of either the CMCP6*lux*Δ*rtxA1* mutant (HG0906) or the CMCP6*lux*Δ*vvhBA* mutant (HG0907) showed no destruction of the villi architecture and infiltration of the lamina propria except only slight swelling (Figure 3E and F). Mice infected with double mutant HG0908 showed no pathology distinct from PBS mock control (Figure. 3G and H). However, the absence of tissue damage cannot be conclusively linked to the toxins by this approach because the bacterial load of wild type in the ileum 8 hours after infection of 10^6^ CFU would be much higher than that of single and double mutant strains due to affects of the loss of cytotoxins on bacterial growth (Figure 2A and B).

**Figure 5:**
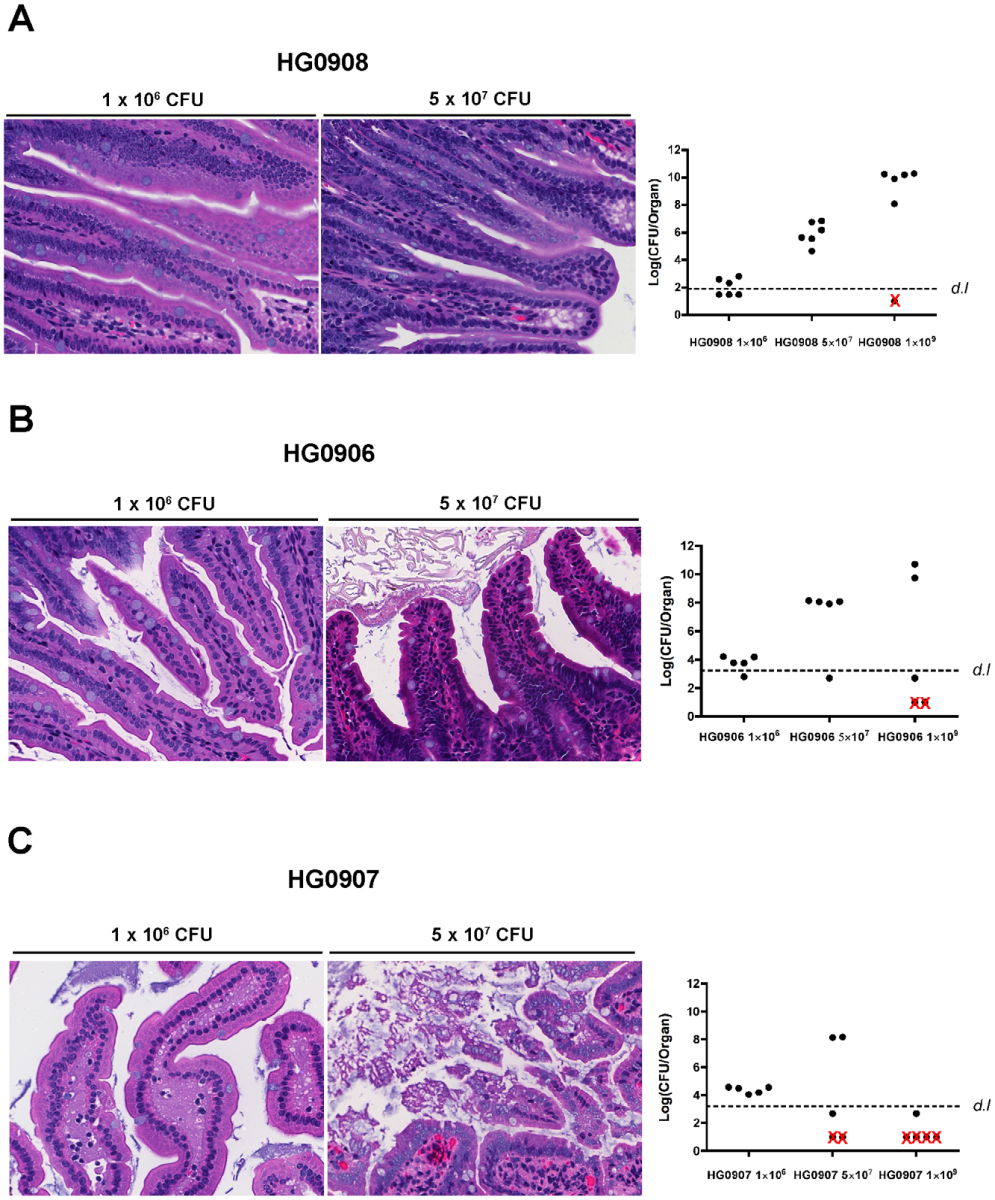
Tissue damage of ileum caused by various doses of single and double mutants. Morphological changes of villi at 8 hr post infection in one representative mouse and bacterial colonization for all mice inoculated with various doses of HG0908 (CMCP6*luxΔrtxA1ΔvvhBA*; A), HG0906 (CMCP6*luxΔrtxA1*; B) and HG0907 (CMCP6*luxΔvvhBA*; C) (magnification 400×). Total recovered CFU from small intestine after removal of 1 cm small intestine for histology are represented as a logarithmic scale, respectively. Values below the dashed line were below the detection limit (*d.l.*) and Red×indicates mice that died before 8 hr.

Therefore, we infected mice with increasing CFU so that the bacterial load in ileum at the point of euthanasia would be equalized. In mice infected with a CMCP6*lux*Δ*rtxA1*Δ*vvhBA* double mutant (HG0908) at a dose of 5×10^7^ or 1×10^9^ CFU (*n* = 6), there was no tissue damage in the ileum of any of the mice (Figure 5A) and the epithelial lining appears similar to the PBS mock infected control group (Figure 3H and I). Notably, even at the dose high enough to kill 1/6 mice in 8 hour, no tissue damage occurred. This finding is significant because it indicates that no other secreted protease or toxin produced by strain CMCP6 is sufficient to cause visible tissue damage in the absence of MARTX_Vv_ or VvhA, even when a concentration of bacteria in the lumen exceeded that normally found for wild type by 8 hr post inoculation.

Staining shows macrophages are present in the lamina propria at low dose infection of double mutant and are secreting only low amounts of IL-1β consistent with the low levels of colonization at 8 hours post inoculation (Figure 4A). By contrast, at a high dose, macrophages are less apparent in the lamina propria and may have moved to the lumen, where high density staining of IL-1β is seen (Figure 4). This focusing of a proinflammatory immune response to the lumen is an effective response since infected mice are surviving a dose that would kill 100% within 8 hr if infected with CMCP6*lux* (HG0905).

### MARTX_Vv_ and VvhA function additively to cause severe tissue damage

To determine whether MARTX_Vv_ and/or VvhA is directly responsible for the tissue damage caused by CMCP6*lux*, similar increasing dose infections were performed with the single toxin deletion strains. When 1×10^9^ CFU of either the isogenic Δ*rtxA1* mutant (*n* = 5) or the Δ*vvhBA* mutant (*n* = 5) was inoculated, 40% or 80% of the mice died within 8 hr post-infection, respectively (Figure 5B and C). The difference in survival compared to the double mutant HG0908 shows that the toxins are able to function independently while the difference between HG0906 and HG0907 further exemplifies the relative import of MARTX_Vv_ over VvhA for overall survival of CMCP6*lux*.

At the intermediate infection dose of 5×10^7^ CFU, the median number of each single mutant recovered from the small intestines were 8.1 and 8.2 Log CFU/organ, respectively (Figure 5B and C); not significantly different from the 7.9 Log CFU/organ recovered from CMCP6*lux* (HG0905) inoculated at only 1×10^6^ CFU. Note that these median values were calculated from surviving, colonized mice that were sacrificed for histopathology and do not include mice that rapidly succumbed to infection or noncolonized mice with recovered CFU below the detection limit. In the CMCP6*lux*Δ*rtxA1* mutant infected mice, only a small portion of the villi showed necrotic epithelial cells and hypercellularity in the lamina propria (Figure 5B) indicating, in the absence of MARTX_Vv_, VvhA induces mild tissue damage accounting for the modest dissemination of this mutant to the liver (Figure 2C). By contrast, samples of intestines from mice infected by the MARTX_Vv_^+^ strain Δ*vvhBA* mutant showed sloughed villi and an infiltration of the lamina propria into the lumen indicating MARTX_Vv_ induces tissue damage that is more severe than associated with VvhA (Figure 5C). This finding is consistent with the significant ability of this mutant to disseminate to the liver (Figure 2C). Importantly, we did not observe the severe tissue destruction similar to that found in the CMCP6*lux* (HG0905) infected group in any of these mice suggesting that both cytotoxins target intestinal epithelial cells and both cause tissue damage that is additive or possibly even synergistic.

## Discussion

Successful and rapid *in vivo* growth of *V. vulnificus* is generally regarded as an essential step in its pathogenesis [11], [26]. We developed a BLI system using the highly virulent *V. vulnificus* strain CMCP6 to directly observe how rapid *in vivo* growth during early infection can influence the outcome of infection. Wild-type *V. vulnificus* CMCP6*lux* infection expanded quickly in mice very early after intestinal infection and all the animals progressed to lethality. By contrast, deletion of one or both of the *rtxA1* and *vvhA* genes led to bacteria with a *in vivo* growth delay leading to reduced CFU in animals by 8–12 hr post infection in the small intestine and other organs, although there was no *in vitro* growth defect (Figure 1). However, while some mice infected with strains missing just one toxin showed little or no growth, many mice still died from infection and others that survived infection emitted a high level of light up to 15 hr (Figure 1A–E). In these mice with detectable light, the slope of light emission representing the growth rate was similar to wild type from first detection to peak infection. Furthermore, co-infection studies revealed the mutant that produces no cytotoxins has the capacity to grow in vivo, but does not have the capacity to alter the host environment to promote its own growth. These findings suggest that the cytolysins cause another phenomenon beyond simply manipulating bacterial load in the animal and that this event is important to cause lethal infection.

Previous studies indicate that dissemination of infection to the liver is a major predictor of mouse mortality after wound infection [9], consistent with clinical reports indicating that hepatic hemorrhage is a frequent cause of death of patients after both wound and food-borne *V. vulnificus* infection [27]–[29]. MARTX_Vv_ and VvhBA from strain CMCP6 are here shown to not only be associated with enhanced *in vivo* growth, but also with necrosis of tissue in the small intestine and translocation of *V. vulnificus* from the small intestine to the liver (Figure 2C). Notably, the small intestine has already been recognized as the site of the most severe tissue necrosis in human autopsy of *V. vulnificus* patients [27].

Although both single toxin gene mutants induced from moderate to severe necrosis and dissemination, the double mutant was completely restricted to the intestine and no damage was evident (Figure 3). Close examination of Figure 1A suggests that light signal from the double mutant occurred predominantly in the lower abdomen compared to wild type light emission from the mid-abdomen, an observation consistent with the ability of the double mutant to grow *in vivo* during transit through the upper and lower bowel, accounting for the increased light signal, but the infection never progressed to the liver.

We next sought to understand if the role of the MARTX_Vv_ in dissemination of CMCP6 during gut infection was to increase the growth rate within the small intestine during the first few hours to create a larger pool of bacteria to express VvhA, proteases or other cytolysins to promote dissemination as proposed by Lo et al. [19] for wound related infections or, if the role of the toxins is to utilize the cytolytic activity to directly lyse intestinal epithelial cells to create a pathway through which the bacteria could disseminate as proposed by Kim et al. [14]. Our study found that, for strain CMCP6, both MARTX_Vv_ and VvhA function additively to cause intestinal tissue necrosis. We also found that *in vivo* growth does occur in the absence of toxins but is restricted to the intestine, possibly to the colon as recovered CFU from the small intestine at 12 hr was decreased compared to wild type despite strong light emission in some animals. Indeed, studies examining wound infection with strain YJ016 also show less dermal tissue damage upon deletion of *rtxA1*, but the effect was negated as secondary to the effect on decreased bacterial load [19]. Using identical inocula, we came to the same conclusions. It was only after we used increased inocula such that bacterial load at the time of euthanasia was equivalent that the role of toxins on tissue necrosis became evident. Thus, it is possible that MARTX_Vv_ and VvhA will be shown to also be involved in tissue necrosis during wound infection, at least for strains CMCP6 and YJ016. However, lethal dose studies have shown that MARTX_Vv_ and VvhA are in general less important to wound induced lethality than i.g. infection [12], [15], [18], [19], [23], supporting the conclusion by Lo et al. [19] that alternate factors have a significant role during wound infection.

While our data support that the cytotoxins target the intestinal epithelium, our results do not negate that the toxins have a significant role in innate immune defense as well. Both *in vitro* and *in vivo*, *V. vulnificus* is also known to cause killing of phagocytic cells [19], [21]. *In vitro*, both toxins are known to induce NLRP3 dependent caspase-1 activation resulting in necrosis of macrophages [21]. The absence of phagocytes in hepatic tissue has been previously noted as a factor that can contribute to patient mortality [30]. However, our study reveals that *V. vulnificus* is inducing massive inflammation, leading to recruitment of monocytes, neutrophils and F4/80-positive macrophages. These results are consistent with an increase of the proinflammatory cytokines TNF-α, IL-6 and IL-1ß that were detected in the sera of *V. vulnificus* septicemic patients [31]. In our study, the increase of IL-1ß secretion in mice inoculated with a lower concentration of CMCP6*lux* suggest that *in vivo*, the action of toxins against macrophages induces pyroptosis, just as it does *in vitro* [21].

A question that remains is whether the toxins are simultaneously promoting inflammation while attempting to keep it at bay by killing the recruited cells. A recent study has revealed that some gut pathogens specifically induce inflammation as a mechanism to promote rapid growth. *Salmonella* is known to induce pyroptosis leading to inflammation [32]. This inflammation then allows *Salmonella* to use tetrathionate respiration in the anaerobic environment of the gut, which promotes bacterial replication and transmission [33], [34]. In the present paper, the tissue damage of villi in small intestine was clearly apparent (Figure 3 and 5) and the inflammation as early as 8 hr post infection is severe (Figure 3D). Thus, while killing of phagocytes is one mechanism that would promote rapid *in vivo* growth, particularly in the bloodstream, it is possible that inflammation itself may promote growth in the intestine. The *ttr* genes that encode the tetrathionate respiration system necessary for *Salmonella* to grow in response to inflammation [34] are present in *V. vulnificus* YJ016 on what appears to be a pathogenicity island [33], [35]. However, the other sequenced *V. vulnificus* strains [36], including CMCP6 used in this study, do not seem to have acquired this island. If the fact that this pathogen induces the host inflammation to promote their own outgrowth is a general consequence, our results suggest that a novel mechanism unrelated, (or in the case of *ttr*+ YJ016 in addition to tetrathionate) is required, depending on the strain isolate.

A final important finding of our work is the evidence that MARTX_Vv_ and VvhBA are directly linked to death of the host. One mechanism that accounts for the linkage to cell death is that the cytotoxins promote movement of the bacteria to bloodstream leading to primary septicemia and septic shock. High bacterial load in the bloodstream and high serum TNFα concentrations have been directly linked to death of patients [37], [38]. In addition to septic shock, the toxins might also contribute to multiorgan failure. This can include necrosis of lung tissue and liver, a common finding in autopsy patients [28], [39]. They could also cause progression of the infections out of the bloodstream into the muscle tissue to cause necrotizing fasciitis, another complication of *V. vulnificus* infections.

Overall, the present study demonstrates that, for *V. vulnificus* isolate CMCP6, MARTX_Vv_, along with VvhBA, performs an essential role during food-borne *V. vulnificus* infection after consumption. These toxins have multiple roles including promotion of rapid *in vivo* growth, destruction of epithelial tissue, causing inflammation through induction of pyroptosis, and possibly causing patient death through tissue destruction in peripheral organs.

Similar studies performed in other *V. vulnificus* clinical isolates will be necessary to determine if findings here with the highly virulent strain CMCP6 will be broadly applicable to all *V. vulnificus* clinical isolates. In particular, we recently found that the predominance of US clinical isolates from patients with primary septicemia (represented by strain MO6-24/O) carry a variant of the MARTX_Vv_ toxin that arose by a recombination in the *rtxA1* gene with the *rtxA* gene from *Vibrio anguillarum*. This recombination likely accounts for an overall 10-fold reduced virulence of MO6-24/O in this animal i.g. infection model compared to CMCP6 [15]. Notably, the loss of a domain of unknown function from the MO6-24/O-type MARTX_Vv_ variant did not affect the function of MARTX_Vv_ as a cytolysin *in vitro* [17] suggesting it would likely retain the ability to induce necrotic tissue damage during intestinal infection.

However, it is possible that the reduced potency of the toxin variant could impact the relative contribution of the toxin to *in vivo* growth and tissue damage such that, perhaps, MARTX_Vv_ and VvhA could be found to have a more equal contribution to intestinal infection by MO6-24/O and similar strains, although we predict that both toxins would continue to have an additive contribution to virulence. Alternatively, the kinetics of infection could be altered such that critical levels of colonization and/or damage will occur later in the infection cycle.

In addition to MO6-24/O and CMCP6-type MARTX_Vv_ variants, other variants of the toxin have been described that have undergone more significant changes by horizontal gene transfer and homologous recombination, including the acquisition of the ability to covalently crosslink actin in epithelial cells [15], [40]. One might predict that these rare variants will have an even more distinct infection profiles when compared to CMCP6. In any event, results in this study have established that both MARTX_Vv_ and VvhA contribute to virulence and provide a baseline for determining if other isolates have similar patterns of disease progression or whether *V. vulnificus* infection develops differently dependent upon the variant of MARTX_Vv_ it expresses.

Finally, while it has been shown here that MARTX_Vv_ and VvhA are critical to infection, these are very likely not the only important virulence factors necessary for intestinal infection. Most notably, *V. vulnificus* Biotype 1 can be separated into two distinct evolutionary lineages: a clinical lineage and environmental lineage [41]. We have recently shown that bacteria from both lineages carry genes for both cytolysins [15], even though strains from the environmental lineage rarely cause clinical infection and are less virulent in mice [9]. Thus, there must be additional *V. vulnificus* factors to define host selection that have yet to be identified and characterized. These would then work in concert with the cytotoxins perhaps by improving growth in the human intestine during infection or by facilitating colonization of the small intestine by interacting with a human epithelial receptor. Regardless of the nature of this other virulence gene, the importance of the cytotoxins cannot be negated since our work demonstrates that the additive destruction of these toxins is essential to disease progression.

## Materials and Methods

### Ethics statement

This study was carried out in strict accordance with the recommendations in the United States Public Health Service (USPHS) regulations and applicable federal and local laws. The protocol (Protocol No. 2009-1016) was approved by the Northwestern University Institutional Animal Care and Use Committee (IACUC) as detailed in methods. All surgery was performed under ketamine-xylazine and isoflurane anesthesia, and all efforts were made to minimize suffering.

### Bacterial strains and growth conditions

The strains and plasmids used in this study are listed in Table 1. *Escherichia coli* strains used for DNA replication or conjugational transfer of plasmids and *Vibrio vulnificus* strains were grown in Luria-Bertani (LB). When appropriate, antibiotics were added to media at the following concentrations: kanamycin (50 µg/ml), rifampicin (50 µg/ml) and chloramphenicol (5 µg/ml). Bacterial growth in LB was monitored using a Beckman DU530 Spectrophotometer.

**Table 1:** Bacterial strains and plasmids used in this study.

Strain or plasmid	Relevant characteristics^a^	Sources or references
**Bacterial Strains**		
*V. vulnificus*		
CMCP6	Clinical isolate; virulent, Rif^r^	P. Gulig
HG0901	CMCP6 with *ΔrtxA1*, Rif^r^	[15]
HG0902	CMCP6 with *ΔvvhBA*, Rif^r^	This study
HG0903	CMCP6 with *ΔrtxA1ΔvvhBA*, Rif^r^	This study
HG0905	CMCP6 with pHGJ1, Rif^r^, Km^r^	This study
HG0906	HG0901 with pHGJ1, Rif^r^, Km^r^	This study
HG0907	HG0902 with pHGJ1, Rif^r^, Km^r^	This study
HG0908	HG0903 with pHGJ1, Rif^r^, Km^r^	This study
HG0909	CMCP6 with pHGJ2, Rif^r^, Km^r^	This study
HG0910	HG0903 with pHGJ2, Rif^r^, Km^r^	This study
*E. coli*		
SM10 λpir	*thi thr leu tonA lacY supE recA*::RP4-2-Tc::Mu λ *pir*; Km^r^; host for π-requiring plasmids; conjugal donor	[46]
S17 λpir	*thi pro hsdR^−^ hsd*M^+^ *recA*::RP4-2-Tc::Mu λ *pir*; Sm^r^; host for π-requiring plasmids; conjugal donor	[47]
**Plasmids**		
pCM17	bioluminescent vector; Km^r^	[24]
pDS132	Suicide vector; *ori*R6K; Cm^r^	[43]
pHGJ1	pCM17 with *oriT*; bioluminescent vector; Km^r^	This study
pHGJ2	pHGJ1 with *ΔluxCDABE*; Km^r^	This study
pHGJ3	*ΔrtxA1* fragment in pDS132; Cm^r^	This study
pHGJ4	*ΔvvhBA* fragment in pDS132; Cm^r^	This study

a , Cm^r^, chloramphenicol resistant; Km^r^, kanamycin resistant; Rif^r^, rifampicin resistant.

### Generation of *V. vulnificus* Δ*rtxA1*, Δ*vvhBA*, Δ*rtxAvvhBA* strains and bioluminescent strains

To inactivate *rtxA1*, *vvhBA* and *rtxA1vvhBA*, overlapping PCR was applied for the construction of *rtxA1* (HG0901), *vvhBA* (HG0902) and *rtxA1vvhBA* (HG0903) deletion mutants [42] (Table 1). The 9635 bp deleted *rtxA1* and the 793 bp deleted *vvhBA* open reading frame (ORF) were ligated with SalI-SacI and XbaI-SacI digested pDS132 [43] forming pHGJ3 and pHGJ4. To generate the *ΔrtxA1* and *ΔvvhBA* mutants by homologous recombination, *E. coli* SM10λ*pir* and S17λ*pir* (containing pHGJ3 and pHGJ4) were used as a conjugal donor to *V. vulnificus* CMCP6 with spontaneous rifampicin resistance. The *ΔrtxA1vvhBA* double mutant was also generated through conjugation of pHGJ3 to HG0902 (Table 1). The conjugation and isolation of the transconjugants were conducted using sucrose counter selection previously described [42].

A pCM17 containing *luxCDABE* and *hok/sok* plasmid [24] was used for generation of bioluminescent *V. vulnificus* strains. To create the conjugatable plasmid, 251 bp of *oriT* DNA from pGP704 was inserted into NheI-SalI digested pCM17 to create pHGJ1. pHGJ1 was then digested with HindIII followed by religation to inactivate the luciferase genes and create pHGJ2 (Table 1). CMCP6*lux* (HG0905) and isogenic *rtxA1* (HG0906), *vvhBA* (HG0907) and *rtxA1vvhBA* (HG0908) mutants were generated by conjugal transfer of pHGJ1 and HG0909 and HG0910 were generated by conjugal transfer of pHGJ2 (Table 1).

### Mouse infection and imaging of bioluminescence from mice

The roles of the *V. vulnificus* CMCP6 MARTX_Vv_ and VvhA in pathogenesis were examined using a mouse model. Female C57BL/6 mice (5–6 weeks old, Harlan, Indianapolis, IN) were individually anesthetized with an i.p. injection of 100 µl of PBS solution containing 10 µg/ml ketamine and 2 µg/ml xylazine per mouse, i.g. inoculated with 50 µl of 1×10^6^ CFU of the indicated *V. vulnificus* strains. Images were acquired using an IVIS 100 (Xenogen Corporation, Alameda, CA). During image acquisition, mice were anesthetized using ketamine-xylazine cocktail at each image cycle. All images were acquired at a preset exposure of 20 sec with medium binning and f/stop = 1 so images could be compared over time. Photons per second emitted by each mouse were quantified and analyzed by defining regions of interest (ROI), using the Living Image 1.0 software. Severely moribund mice unlikely to survive to the next imaging cycle were euthanized after imaging and counted as non-survivors.

### Histology and immunohistochemistry

To observe the tissue damage in mice small intestine, infected mice were sacrificed at specific time points and 1 cm of ileum immediately adjacent to the cecal-ileal junction was fixed by 10% neutral phosphate buffered formaldehyde solution (Sigma, St. Louis, MO) for 16 hr. Histopathology was performed at the Northwestern University Pathology Core Facility. The ileum was embedded in paraffin, and stained with hematoxylin and eosin (H&E).

A single immunohistochemical staining procedure was performed to characterize the necrotized cells and to detect the cytokines secretion. Briefly, tissue sections were placed in a 60°C oven overnight for tissue to adhere. The sections were dewaxed in xylene, rehydrated through graded alcohols to water. Antigen retrieval was done by placing the slides in citrate buffer and pressure cooked up to 125°C for 30 sec and gradually reduced to 90°C over 40 min. Slides were then cooled down at room temperature for 20 min and placed in DAKO butter (DAKO, Carpineteria, CA). Then immunostaining for ß–catenin, CD45, F4/80, and IL-1ß was performed on a DAKO Autostainer Plus using a DAKO Envision system (DAKO). Sections were first quenched with hydrogen peroxide (H_2_O_2_) for 10 min and incubated with primary antibodies for 60 min. Primary rabbit polyclonal antibodies to ß-catenin, CD45 and IL-1ß (Abcam, Cambridge, UK), at 1∶50 dilution, and rat monoclonal antibodies to F4/80 (Abcam), at a 1∶100 dilution, were used. Secondary antibodies were applied at a dilution of 1∶200 for 30 min, followed by incubation with polymer link streptavidin-horseradish peroxidase (HRP) reagent and 3,3′-diaminobenzidine (DAB; DAKO). The slides were counter-stained with blue Mayer's Hematoxylin and primary antibodies were omitted in negative controls. Then the stained slides were photographed using a Zeiss Axioskop (MicroImaging, Thornwood, NY) microscope with Nuance spectral camera (CRI, Woburn, Mass).

### Determination of bacterial colonization in mice organs and ELISA for mice cytokine in small intestine

Mouse colonization assays were performed essentially as described earlier for *Vibrio cholerae* infection [44]. Briefly, five C57BL/6 female mice (5–6 weeks old, Harlan, Indianapolis, IN) per each group were euthanized by cervical dislocation under anesthesia at 8 or 12 hr after inoculation of the indicated *V. vulnificus* strains or PBS. After terminal ileum dissection for histology, the liver, spleen and remaining small intestine were dissected. Then it was homogenized in 5 ml (small intestine and liver) or 3 ml (spleen) of PBS and serially diluted for plate counts of recovered colony forming units (CFU) on LB plate containing rifampicin. Mice for which fewer than 10 colonies were recovered from 50 µl of the homogenated extract were plotted below the detection limit line. Recovery of bacteria is reported as a Colonization Index (Col. Ind.) calculated as CFU_recovered_/CFU_inoculated_ or CFU/organ at logarithmic scale. The remaining homogenates of small intestine was centrifuged at 13400×*g* for 5 min at 4°C and the supernatant were kept at −80°C. The supernatants were thawed on ice immediately prior to assay. IL-1ß levels in small intestines were determined from homogenated extracts by ELISA (Enzyme-linked immunosorbent assay, BioLegend, San Diego, CA) kits according to manufacturer's instruction.

### Virulence determination and cytotoxicity assay of *lux*+ *V. vulnificus* strains

Virulence of *lux*+ *V. vulnificus* strains was determined in a morbidity assay as previously described [15] and the LD_50_ for each strain was calculated by the method of Reed and Muench [45].

To examine the cytotoxicity of *lux*+ *V. vulnificus* strains, the HeLa cells were grown in Dulbecco's modified Eagle's medium containing 10% fetal bovine serum and seeded in 12 well culture plates to a density of 8.5×10^5^ cells per well. After growing overnight at 37°C in 5% CO_2_, the monolayer of HeLa cells were infected with *lux*+ *V. vulnificus* strains at a multiplicity of infection of 25 and the cytotoxicity was then determined by measuring the activity of LDH in the supernatant at 1 to 5 hr post-infection using a CytoTox 96 Non-Radioactive Cytotoxicity Assay Kit (Promega, Madison, WI) according to manufacturer's instructions.

### Statistical analysis

All data were graphed and analyzed using GraphPad Prism 4 for MacIntosh Software (San Diego, CA). Statistical significance for LDH assays, growth curve, and ELISA assays was determined in pairwise comparisons using a student t-test. A Mann-Whitney non-parametric t-test comparing means was used for mouse colonization studies. Significance of survival curves was determined using the log-rank test.

## Supporting Information

Figure S1 **The effect of luciferase-expressing** ***V. vulnificus*** **in survival proportions of mice and** ***in vitro*** **growth kinetics.** (A) 5–6 weeks old C57BL/6 mice were infected with CMCP6, HG0905 (CMCP6*lux*) and HG0909 (*lux*- CMCP6) strains i.g. and survival proportions of mice were compared. (B) Cultures of CMCP6 and HG0905 were grown in LB broth at 30°C (*, *p*<0.05; **, *p*<0.01).
(TIF)

Figure S2 **Effect of MARTX_Vv_ and VvhA in** ***lux*** **+** ***V. vulnificus*** **on HeLa cell lysis activity.** HeLa cells were infected with the HG0905, HG0906, HG0907 and HG0908 at MOI of 25 and LDH activity were determined at various incubation times (*, *p*<0.05; **, *p*<0.01).
(TIF)

Table S1 **Lethality of luciferase-expressing** ***V.vulnificus*** **strains to mice.**
(DOCX)

## References

[1] Blake PA, Merson MH, Weaver RE, Hollis DG, Heublein PC (1979). Disease caused by a marine *Vibrio*. Clinical characteristics and epidemiology.. N Engl J Med.

[2] Linkous DA, Oliver JD (1999). Pathogenesis of *Vibrio vulnificus*.. FEMS Microbiol Lett.

[3] Strom MS, Paranjpye RN (2000). Epidemiology and pathogenesis of *Vibrio vulnificus*.. Microbes Infect.

[4] Bowdre JH, Poole MD, Oliver JD (1981). Edema and hemoconcentration in mice experimentally infected with *Vibrio vulnificus*.. Infect Immun.

[5] Jones MK, Oliver JD (2009). *Vibrio vulnificus*: disease and pathogenesis.. Infect Immun.

[6] Chiang SR, Chuang YC (2003). *Vibrio vulnificus* infection: clinical manifestations, pathogenesis, and antimicrobial therapy.. J Microbiol Immunol Infect.

[7] Horseman MA, Surani S (2011). A comprehensive review of *Vibrio vulnificus*: an important cause of severe sepsis and skin and soft-tissue infection.. Int J Infect Dis.

[8] Tacket CO, Brenner F, Blake PA (1984). Clinical features and an epidemiological study of *Vibrio vulnificus* infections.. J Infect Dis.

[9] Thiaville PC, Bourdage KL, Wright AC, Farrell-Evans M, Garvan CW (2011). Genotype is correlated with but does not predict virulence of *Vibrio vulnificus* biotype 1 in subcutaneously inoculated, iron dextran-treated mice.. Infect Immun.

[10] Daniels NA (2011). *Vibrio vulnificus* oysters: pearls and perils.. Clin Infect Dis.

[11] Gulig PA, Bourdage KL, Starks AM (2005). Molecular Pathogenesis of *Vibrio vulnificus*.. J Microbiol.

[12] Fan JJ, Shao CP, Ho YC, Yu CK, Hor LI (2001). Isolation and characterization of a *Vibrio vulnificus* mutant deficient in both extracellular metalloprotease and cytolysin.. Infect Immun.

[13] Chung KJ, Cho EJ, Kim MK, Kim YR, Kim SH (2010). RtxA1-induced expression of the small GTPase Rac2 plays a key role in the pathogenicity of *Vibrio vulnificus*.. J Infect Dis.

[14] Kim YR, Lee SE, Kook H, Yeom JA, Na HS (2008). *Vibrio vulnificus* RTX toxin kills host cells only after contact of the bacteria with host cells.. Cell Microbiol.

[15] Kwak JS, Jeong HG, Satchell KJ (2011). *Vibrio vulnificus rtxA1* gene recombination generates toxin variants with altered potency during intestinal infection.. Proc Natl Acad Sci U S A.

[16] Kim JR, Oh DR, Cha MH, Pyo BS, Rhee JH (2008). Protective effect of polygoni cuspidati radix and emodin on *Vibrio vulnificus* cytotoxicity and infection.. J Microbiol.

[17] Lee JH, Kim MW, Kim BS, Kim SM, Lee BC (2007). Identification and characterization of the *Vibrio vulnificus rtxA* essential for cytotoxicity in vitro and virulence in mice.. J Microbiol.

[18] Liu M, Alice AF, Naka H, Crosa JH (2007). The HlyU protein is a positive regulator of *rtxA1*, a gene responsible for cytotoxicity and virulence in the human pathogen *Vibrio vulnificus*.. Infect Immun.

[19] Lo HR, Lin JH, Chen YH, Chen CL, Shao CP (2011). RTX toxin enhances the survival of *Vibrio vulnificus* during infection by protecting the organism from phagocytosis.. J Infect Dis.

[20] Shinoda S, Miyoshi S, Yamanaka H, Miyoshi-Nakahara N (1985). Some properties of *Vibrio vulnificus* hemolysin.. Microbiol Immunol.

[21] Toma C, Higa N, Koizumi Y, Nakasone N, Ogura Y (2010). Pathogenic *Vibrio* activate NLRP3 inflammasome via cytotoxins and TLR/nucleotide-binding oligomerization domain-mediated NF-kappa B signaling.. J Immunol.

[22] Yamamoto K, Wright AC, Kaper JB, Morris JG (1990). The cytolysin gene of *Vibrio vulnificus*: sequence and relationship to the *Vibrio cholerae* E1 Tor hemolysin gene.. Infect Immun.

[23] Wright AC, Morris JG (1991). The extracellular cytolysin of *Vibrio vulnificus*: inactivation and relationship to virulence in mice.. Infect Immun.

[24] Morin CE, Kaper JB (2009). Use of stabilized luciferase-expressing plasmids to examine in vivo-induced promoters in the *Vibrio cholerae* vaccine strain CVD 103-HgR.. FEMS Immunol Med Microbiol.

[25] Pineyro P, Zhou X, Orfe LH, Friel PJ, Lahmers K (2010). Development of two animal models to study the function of *Vibrio parahaemolyticus* type III secretion systems.. Infect Immun.

[26] Starks AM, Bourdage KL, Thiaville PC, Gulig PA (2006). Use of a marker plasmid to examine differential rates of growth and death between clinical and environmental strains of *Vibrio vulnificus* in experimentally infected mice.. Mol Microbiol.

[27] Chen Y, Satoh T, Tokunaga O (2002). *Vibrio vulnificus* infection in patients with liver disease: report of five autopsy cases.. Virchows Arch.

[28] Shirouzu K, Miyamoto Y, Yasaka T, Matsubayashi Y, Morimatsu M (1985). *Vibrio vulnificus* septicemia.. Acta Pathol Jpn.

[29] Tajiri T, Tate G, Akita H, Ohike N, Masunaga A (2008). Autopsy cases of fulminant-type bacterial infection with necrotizing fasciitis: group A (beta) hemolytic *Streptococcus pyogenes* versus *Vibrio vulnificus* infection.. Pathol Int.

[30] Hor LI, Chang TT, Wang ST (1999). Survival of *Vibrio vulnificus* in whole blood from patients with chronic liver diseases: association with phagocytosis by neutrophils and serum ferritin levels.. J Infect Dis.

[31] Shin SH, Shin DH, Ryu PY, Chung SS, Rhee JH (2002). Proinflammatory cytokine profile in *Vibrio vulnificus* septicemic patients' sera.. FEMS Immunol Med Microbiol.

[32] Fink SL, Cookson BT (2007). Pyroptosis and host cell death responses during *Salmonella* infection.. Cell Microbiol.

[33] Winter SE, Baumler AJ (2011). A breathtaking feat: to compete with the gut microbiota, *Salmonella* drives its host to provide a respiratory electron acceptor.. Gut Microbes.

[34] Winter SE, Thiennimitr P, Winter MG, Butler BP, Huseby DL (2010). Gut inflammation provides a respiratory electron acceptor for *Salmonella*.. Nature.

[35] Chen CY, Wu KM, Chang YC, Chang CH, Tsai HC (2003). Comparative genome analysis of *Vibrio vulnificus*, a marine pathogen.. Genome Res.

[36] Gulig PA, de Crecy-Lagard V, Wright AC, Walts B, Telonis-Scott M (2010). SOLiD sequencing of four *Vibrio vulnificus* genomes enables comparative genomic analysis and identification of candidate clade-specific virulence genes.. BMC Genomics.

[37] Kim DM, Jung SI, Jang HC, Lee CS, Lee SH (2011). *Vibrio vulnificus* DNA load and mortality.. J Clin Microbiol.

[38] Lee JY, Kim DM, Yun NR, Neupane GP, Jung SI (2011). Tumor necrosis factor-alpha and mortality in patients infected with *Vibrio vulnificus*.. Am J Trop Med Hyg.

[39] Wongpaitoon V, Sathapatayavongs B, Prachaktam R, Bunyaratvej S, Kurathong S (1985). Spontaneous *Vibrio vulnificus* peritonitis and primary sepsis in two patients with alcoholic cirrhosis.. Am J Gastroenterol.

[40] Roig FJ, Gonzalez-Candelas F, Amaro C (2011). Domain organization and evolution of multifunctional autoprocessing repeats-in-toxin (MARTX) toxin in *Vibrio vulnificus*.. Appl Environ Microbiol.

[41] Cohen AL, Oliver JD, DePaola A, Feil EJ, Boyd EF (2007). Emergence of a virulent clade of *Vibrio vulnificus* and correlation with the presence of a 33-kilobase genomic island.. Appl Environ Microbiol.

[42] Fullner KJ, Mekalanos JJ (1999). Genetic characterization of a new type IV-A pilus gene cluster found in both classical and El Tor biotypes of *Vibrio cholerae*.. Infect Immun.

[43] Philippe N, Alcaraz JP, Coursange E, Geiselmann J, Schneider D (2004). Improvement of pCVD442, a suicide plasmid for gene allele exchange in bacteria.. Plasmid.

[44] Olivier V, Queen J, Satchell KJ (2009). Successful small intestine colonization of adult mice by *Vibrio cholerae* requires ketamine anesthesia and accessory toxins.. PLoS One.

[45] Reed L, Muench H (1938). A simple method of estimating fifty percent endpoints.. Amer J Hygiene.

[46] Miller VL, Mekalanos JJ (1988). A novel suicide vector and its use in construction of insertion mutations: osmoregulation of outer membrane proteins and virulence determinants in *Vibrio cholerae* requires toxR.. J Bacteriol.

[47] Simon R, Priefer U, Puhler A (1983). A Broad Host Range Mobilization System for Invivo Genetic-Engineering - Transposon Mutagenesis in Gram-Negative Bacteria.. Bio-Technology.

