# Correction: Additive Function of *Vibrio vulnificus* MARTX _Vv_ and VvhA Cytolysins Promotes Rapid Growth and Epithelial Tissue Necrosis During Intestinal Infection

**DOI:** 10.1371/journal.ppat.1007895

**Published:** 2019-06-19

**Authors:** Hee-Gon Jeong, Karla J. F. Satchell

The genetic designation of the strain HG0902 is incorrect in the Results section and the Materials and Methods section. It should be CMCP6*rif* Ω*vvhA*::*bla*, *Rif* ^*r*^. The authors have provided the following text to replace the third, fourth, and fifth paragraphs of the Materials and Methods section to clarify this error:

## Generation of *V*. *vulnificus* Δ*rtxA1*, Δ*vvhA*, Δ*rtxAvvhA* strains and bioluminescent strains

To inactivate *vvhA*, the gene was amplified, captured in pCR-TOPO-Blunt and then digested with HpaI to remove 972 bp. The Δ*vvhA* fragment was moved to pWM91 and the resulting plasmid transformed to *E*. *coli* SM10 λ*pir* for conjugation to *V*. *vulnificus* CMCP6. Subsequent sucrose counter selection as previously described [44] resulted in incomplete resolution of the integrating plasmid such that resulting strain HG0902 has *vvhA* disrupted by plasmid integration and retains ampicillin resistance associated with the plasmid.

To inactivate *rtxA1*, a fragment of *rtxA1* generated by overlapping PCR with 9635 bp removed was ligated with SalI-SacI and XbaI-SacI digested pDS132 [45] forming pHGJ3. To generate the *ΔrtxA1* mutant, the plasmid was transformed to *E*. *coli* S17λ*pir* (containing pHGJ3 and the plasmid with the Δ*vvhA* fragment) were used as a conjugal donor to *V*. *vulnificus*. The *ΔrtxA1vvhA* double mutant was also generated through conjugation of pHGJ3 to HG0902 (Table 1).

**Table 1:** Bacterial strains and plasmids used in this study.

Strain or Plasmid	Relevant characteristics ^a^	Sources or Reference
**Bacterial Strains**		
*V*. *vulnificus*		
CMCP6	Clinical isolate; virulent, Rif ^r^	P. Gulig
HG0901	CMCP6 *ΔrtxA1*, Rif^r^	[16]
HG0902	CMCP6 Ω*vvhA*::*bla (ΔvvhA)*, Rif^r^, Ap^r^	This study
HG0903	HG0902 *ΔrtxA1 (ΔrtxAvvhA)*, Rif^r^, Ap^r^	This study
HG0905	CMCP6 with pHGJ1, Rif^r^, Km^r^	This study
HG0906	HG0901 with pHGJ1, Rif^r^, Km^r^	This study
HG0907	HG0902 with pHGJ1, Rif^r^, Km^r^, Ap^r^	This study
HG0908	HG0903 with pHGJ1, Rif^r^, Km^r^, Ap^r^	This study
HG0909	CMCP6 with pHGJ2, Rif^r^, Km^r^	This study
HG0910	HG0903 with pHGJ2, Rif^r^, Km^r^, Ap^r^	This study
*E*. *coli*		
SM10 λpir	*thi thr leu tonA lacY supE recA*::RP4-2*-* Tc::Mu λ*pir*; Km^r^; host for π-requiring plasmids; conjugal donor	[48]
S17 λpir	*thi pro hsdR*^*-*^ *hsd*M^+^ *recA*::RP4-2*-*Tc::Mu λ*pir* Sm^r^; host for π-requiring plasmids; conjugal donor	[49]
**Plasmids**		
pCM17	bioluminescent vector; Km^r^	[25]
pHGJ1	pCM17 with *oriT*; bioluminescent vector, Km^r^	This study
pHGJ2	pHGJ1 with *ΔluxCDABE*; Km^r^	This study
pHGJ3	*ΔrtxA1* fragment in pDS132 [45]; Cm^r^	This study
pΔ*vvhA*-pWM91	*ΔvvhA* fragment in pWM91 [44]; Ap^r^	This study

^a^, Cm^r^, chloramphenicol resistant; Km^r^, kanamycin resistant; Rif^r^, rifampicin resistant.

A pCM17 containing *luxCDABE* and *hok/sok* plasmid [25] was used for generation of bioluminescent *V*. *vulnificus* strains. To create the conjugatable plasmid, 251 bp of *oriT* from pGP704 was inserted into NheI-SalI digested pCM17 to create pHGJ1. pHGJ1 was then digested with HindIII followed by elegation to inactivate the luciferase genes and create pHGJ2 (Table 1). CMCP6*lux* (HG0905) and isogenic *rtxA1* (HG0906), *vvhA* (HG0907) and *rtxA1vvhA* (HG0908) mutants were generated by conjugal transfer of pHGJ1 and HG0909 and HG0910 were generated by conjugal transfer of pHGJ2 (Table 1).

As a result of the incorrectly labelled strain, there are also errors in the legends for Figs 1–5. Please see the corrected Figure Legends here.

**Fig 1:**
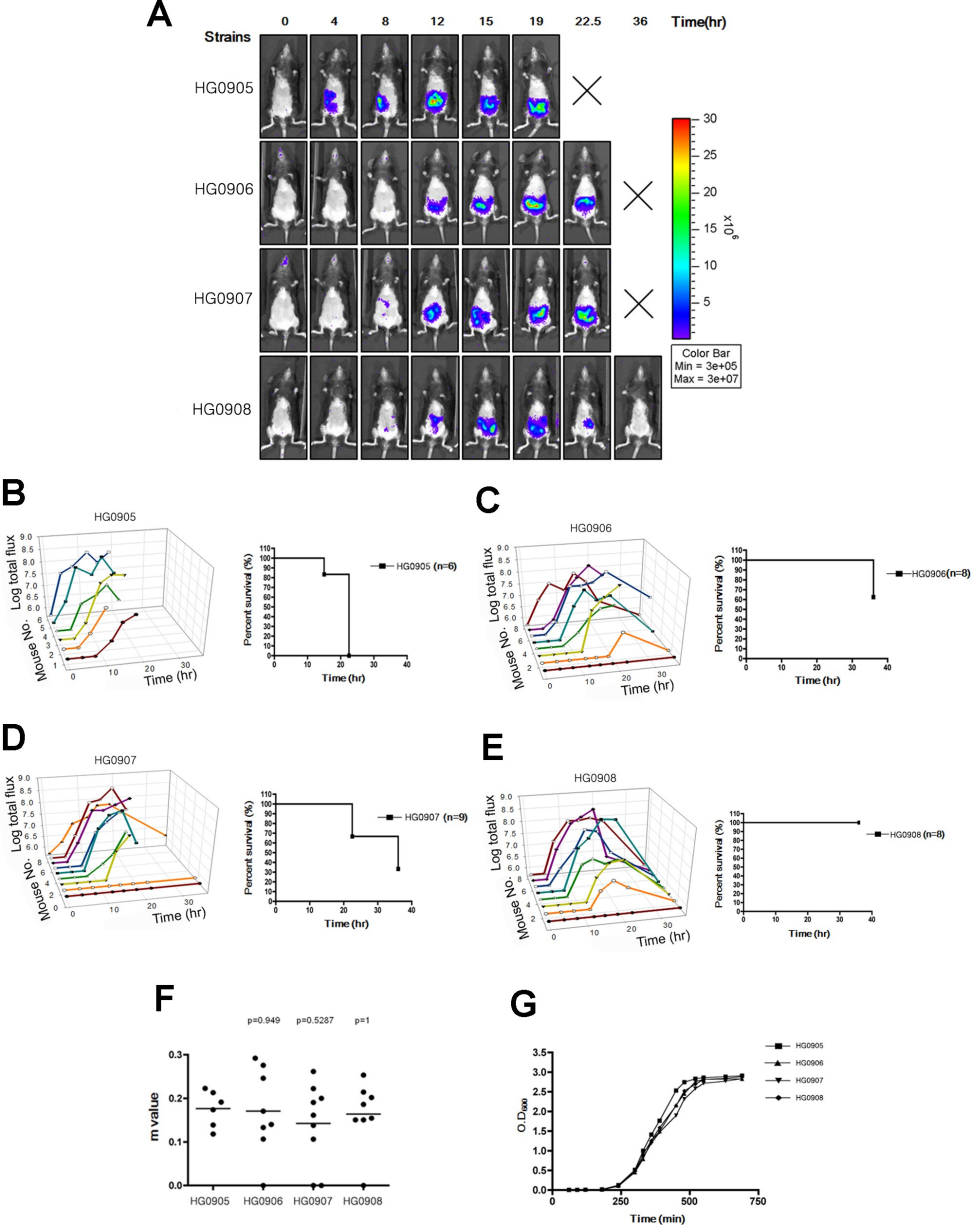
Bioluminescence quantification and lethality measurement in mice infected with *V*. *vulnificus* strains containing pHGJ1. (A) 5–6 weeks old C57BL/6 mice were infected with 1 x 10^6^ CFU of HG0905 (CMCP6*lux*) and isogenic mutants HG0906 (*ΔrtxA1*), HG0907 (*ΔvvhA*) or HG0908 (*ΔrtxA1vvhA*) strains i.g. as indicated. Representative images were acquired at 0, 4, 8, 12, 15, 19, 22.5 and 36 hr post infection, and the color scale of total flux represents the photons s^-1^ cm^-2^ sr^-1^ for combined images is shown. (B-E) Survival curve and light emission from every individual mouse infected with HG0905 (B), HG0906 (C), HG0907 (D) and HG0908 (E) were measured. (F) Slopes (m) between onset and peak point from total flux of individual mice were measured. (G) *In vitro* growth curves of strains in LB broth at 30°C with shaking of HG0905, HG0906, HG0907 and HG0908 for OD_600_.

**Fig 2:**
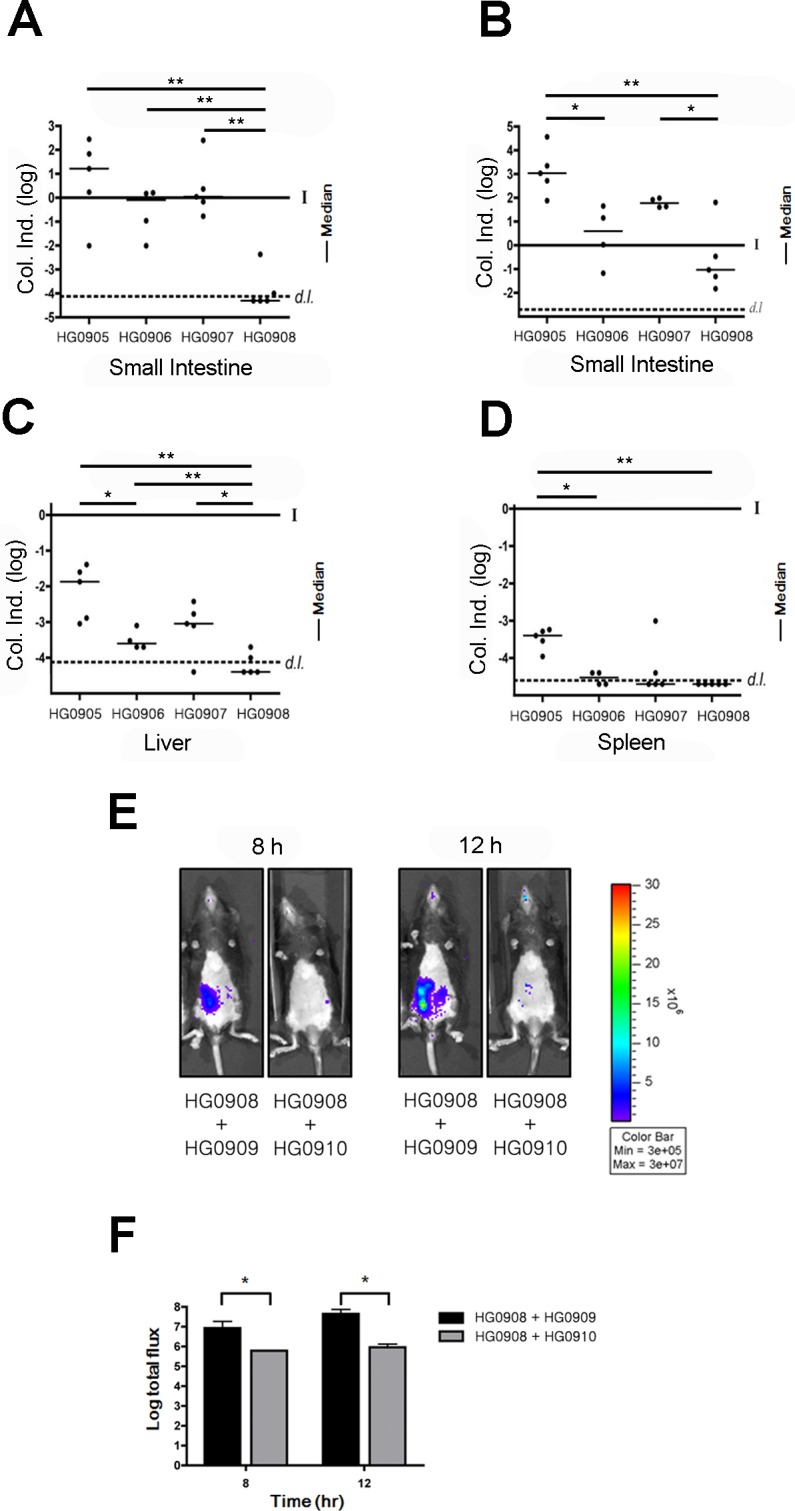
Dynamics of bacterial colonization and dissemination. C57BL/6 mice (5–6 weeks old) were infected with 1 x 10^6^ CFU of HG0905 (CMCP6*lux*) and isogenic mutants HG0906 (*ΔrtxA1*), HG0907 (*ΔvvhA*) and HG0908 (*ΔrtxA1vvhA*). Small intestines after 8 hr (A) and 12 hr (B) post infection, liver (C) and spleen (D) were collected, homogenized and plated for CFU counting (*, *p* < 0.05; **, *p* < 0.01). Values are reported as a log colonization index (Col. Ind.), which is defined as the log of the number of recovered CFU divided by the number of input CFU. Solid line at 0 indicates that CFU recovered was identical to the input CFU (I) while values above the line signify growth and values below the line indicate clearance. Values below the dashed line (*d*.*l*.) were below the detection limit. (E) Co-infections experiments were performed by inoculation of a mouse with the bacterial suspension prepared by mixing equal numbers of the HG0908 (*lux*+ *ΔrtxA1vvhA*) and HG0909 (*lux*^-^ CMCP6), and also HG0908 and HG0910 (*lux*^-^ *ΔrtxA1vvhA*). BLI from mice were acquired after 8 hr and 12 hr. And (F) median values of total flux were also quantified.

**Fig 3:**
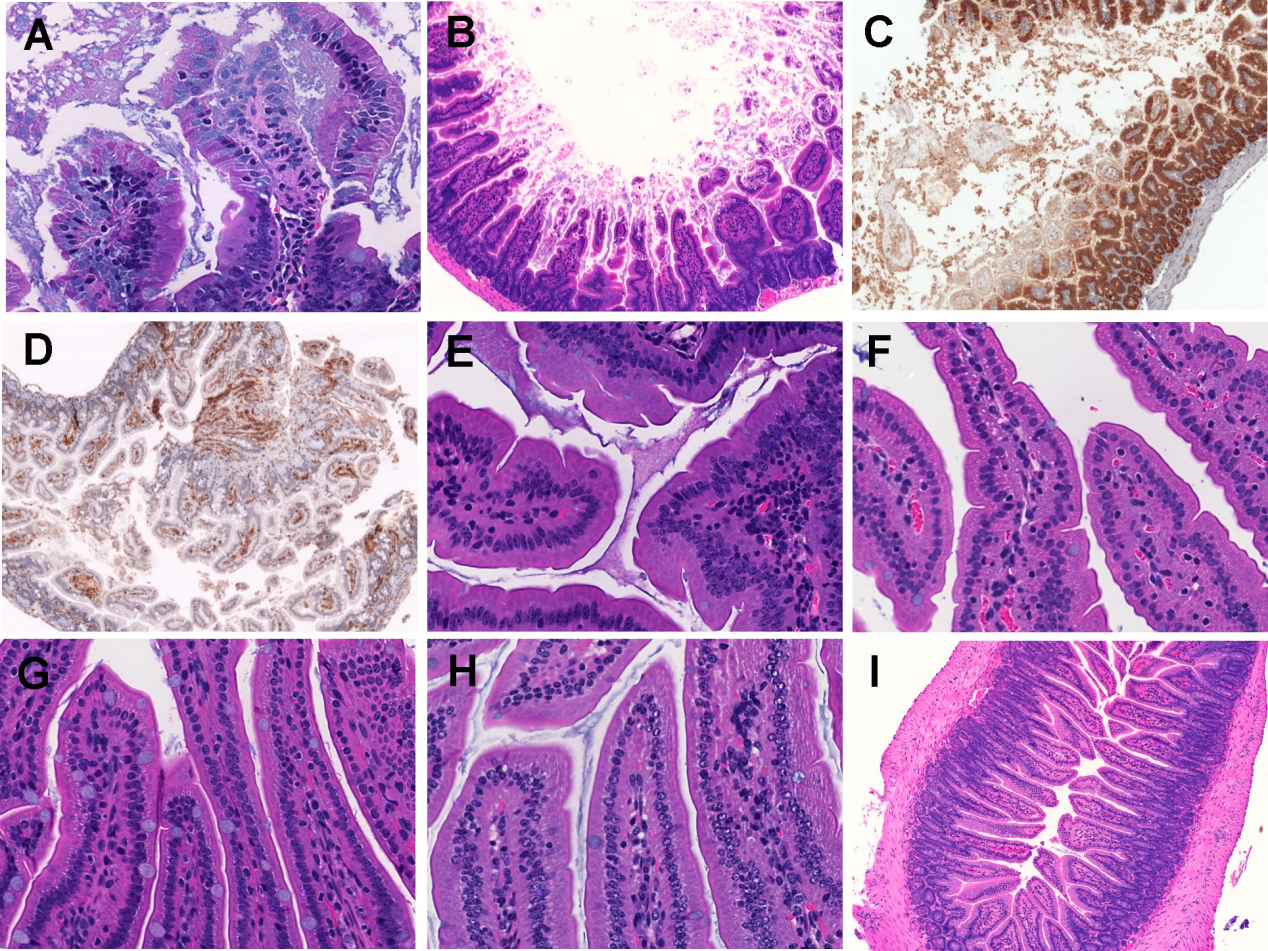
Histopathological examination of mice small intestine inoculated with *V*. *vulnificus* strains. Micrographs of ileum of mice inoculated i.g. with 1 x 10^6^ CFU of HG0905 (CMCP6*lux*; A to D) and isogenic mutants HG0906 (*ΔrtxA1*; E), HG0907 (*ΔvvhA*; F), or HG0908 (*ΔrtxA1vvhA*; G). Mice ileum infected with PBS were used for the negative control (H and I). (A) Infiltration of lamina propria in mice ileum infected with HG0905. Sloughed villi, necrotic debris epithelial cells and leukocyte are abundant in lumen, which are stained by H&E (B), anti-ß-catenin antibody (brown) (C) and anti-CD45 antibody (brown) (D). Both magnified views of villi infected with HG0906 (E) and HG0907 (F) showed a little swelling. (G) Intact villi were observed from mice infected with HG0908 (magnification of A, E, F, G and H, 400x; magnification of B, C, D and I, 200x).

**Fig 4:**
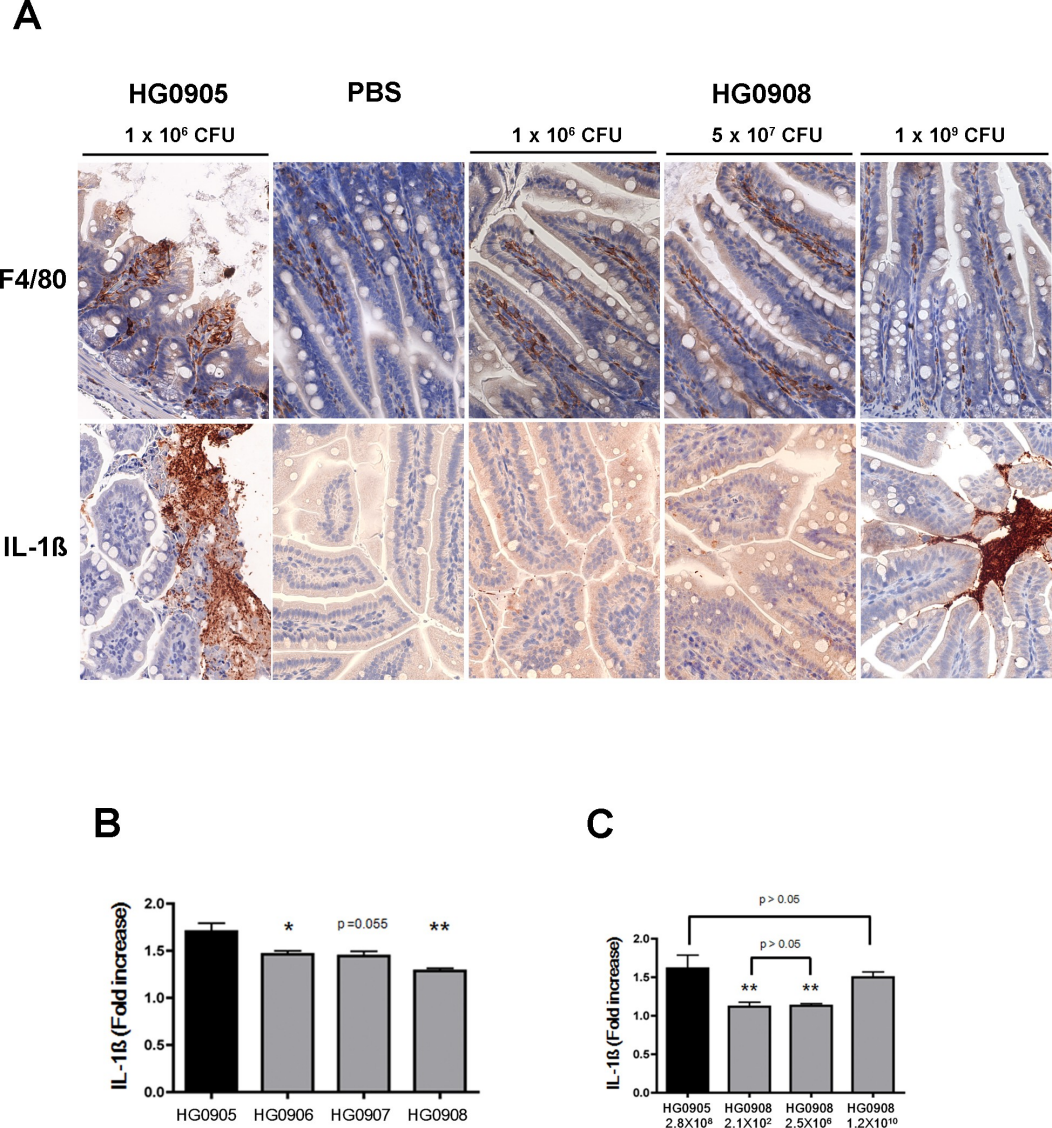
Tissue damage caused by various doses of HG0906 and HG0907 at mice ileum. Morphological changes of villi at 8 hr post infection in one representative mouse and bacterial colonization for all mice inoculated with various doses of HG0906 (CMCP6*luxΔrtxA1*; A) and HG0907 (CMCP6*luxΔvvhA*; B). Total recovered CFU from small intestine after removal of 1 cm small intestine for histology are represented as a logarithmic scale, respectively. Values below the dashed line were below the detection limit (*d*.*l*.) and Red x indicates mice that died before 8 hr. (A) At a lower infection dose of 1 x 10^6^ CFU of HG0906, almost intact villi were observed. Swelling and a little necrosis of villi were shown at a medium infection dose of 5 x 10^7^ CFU magnification 400x). (B) Comparing to the same dose of HG0906, there were no difference in villi morphology infected with HG0907, while small intestine infected with 1 x 10^6^ CFU of HG0907 showed necrosis and infiltration of lamina propria (magnification 400x).

**Fig 5:**
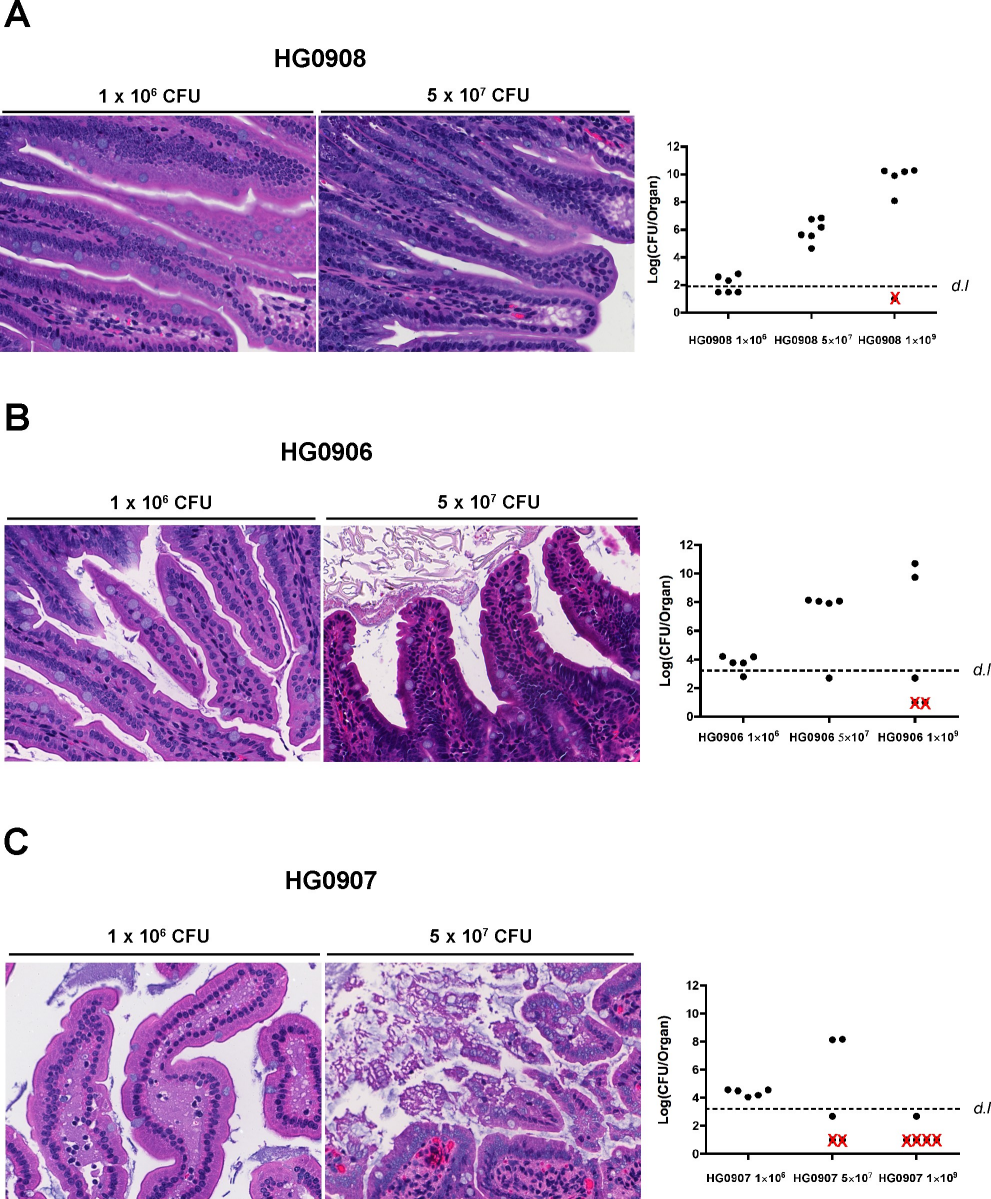
Comparison of HG0908 effect on villi, macrophage and IL-1ß expression to HG0905. (A) H&E staining of ileum (magnification 400x from representative mouse and colonization of all mice in experiment when inoculated with various doses of HG0908 (CMCP6*luxΔrtxA1vvhA*). Red x indicates mouse that died before 8 hr. (B) Immunohistochemistry staining of ileum (magnification 400x) for F4/80 antibody (brown) and IL-1ß antibody (brown) in mice infected with 1 x 10^6^ CFU of HG0905 (CMCP6*lux*) or various doses of HG0908. (C and D) Fold increase of IL-1ß secretion over PBS control in small intestine from mice infected with (C) 1 x 10^6^ CFU of indicated strain or (D) varying concentrations of HG0905 or HG0908 as indicated were measured by ELISA after 8 hr infection (*, *p* < 0.05; **, *p*< 0.01).

As a result of the incorrectly labelled strain, there are also errors in Table 1. Please see the corrected Table 1 here.

The authors have provided a tracked changes version of the manuscript to provide context for these changes.

## Supporting information

S1 File Tracked changes version of the manuscript. (DOCX)
