# Supplementary figures and images for: Additive Function of Vibrio vulnificus MARTXVv and VvhA Cytolysins Promotes Rapid Growth and Epithelial Tissue Necrosis During Intestinal Infection
Source: PLoS Pathog. 2012 Mar 22;8(3):e1002581. doi: 10.1371/journal.ppat.1002581 (PMC3310748; doi:10.1371/journal.ppat.1002581)

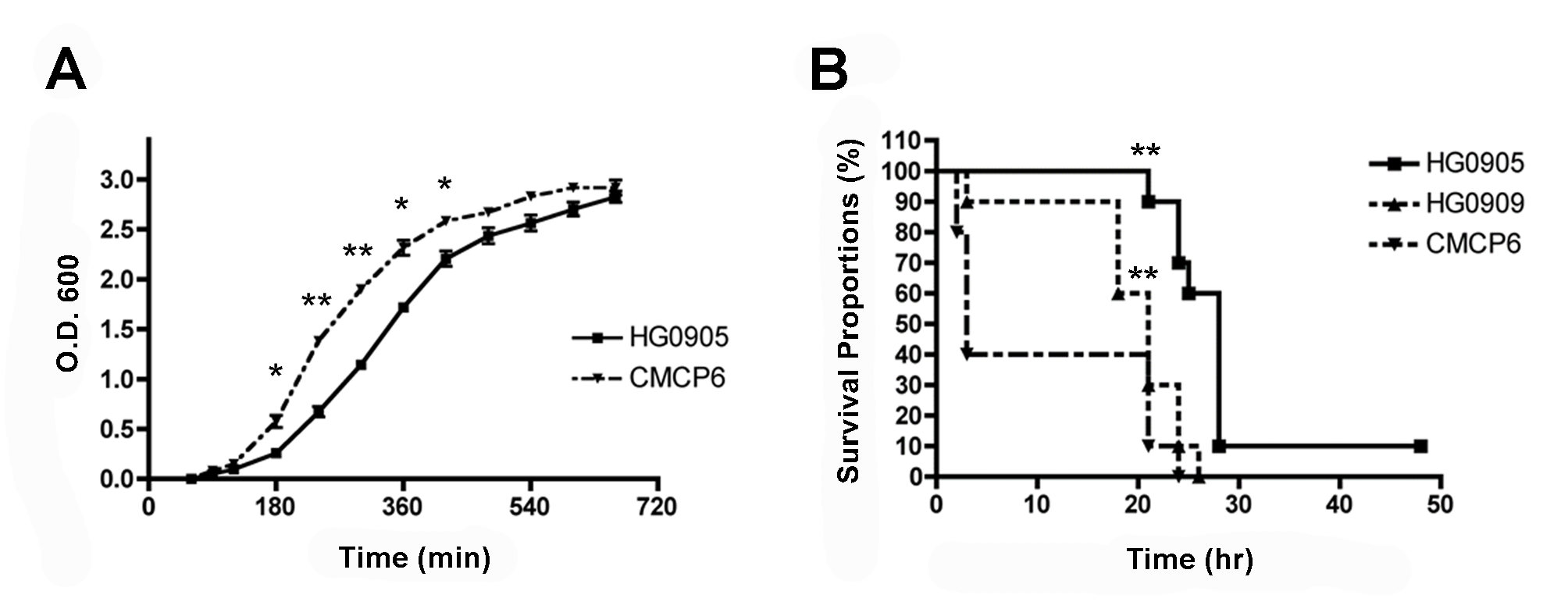

Supplement: Figure S1 — The effect of luciferase-expressing V. vulnificus in survival proportions of mice and in vitro growth kinetics. (A) 5–6 weeks old C57BL/6 mice were infected with CMCP6, HG0905 (CMCP6lux) and HG0909 (lux- CMCP6) strains i.g. and survival proportions of mice were compared. (B) Cultures of CMCP6 and HG0905 were grown in LB broth at 30°C (*, p<0.05; **, p<0.01). (TIF) [file ppat.1002581.s001.tif]

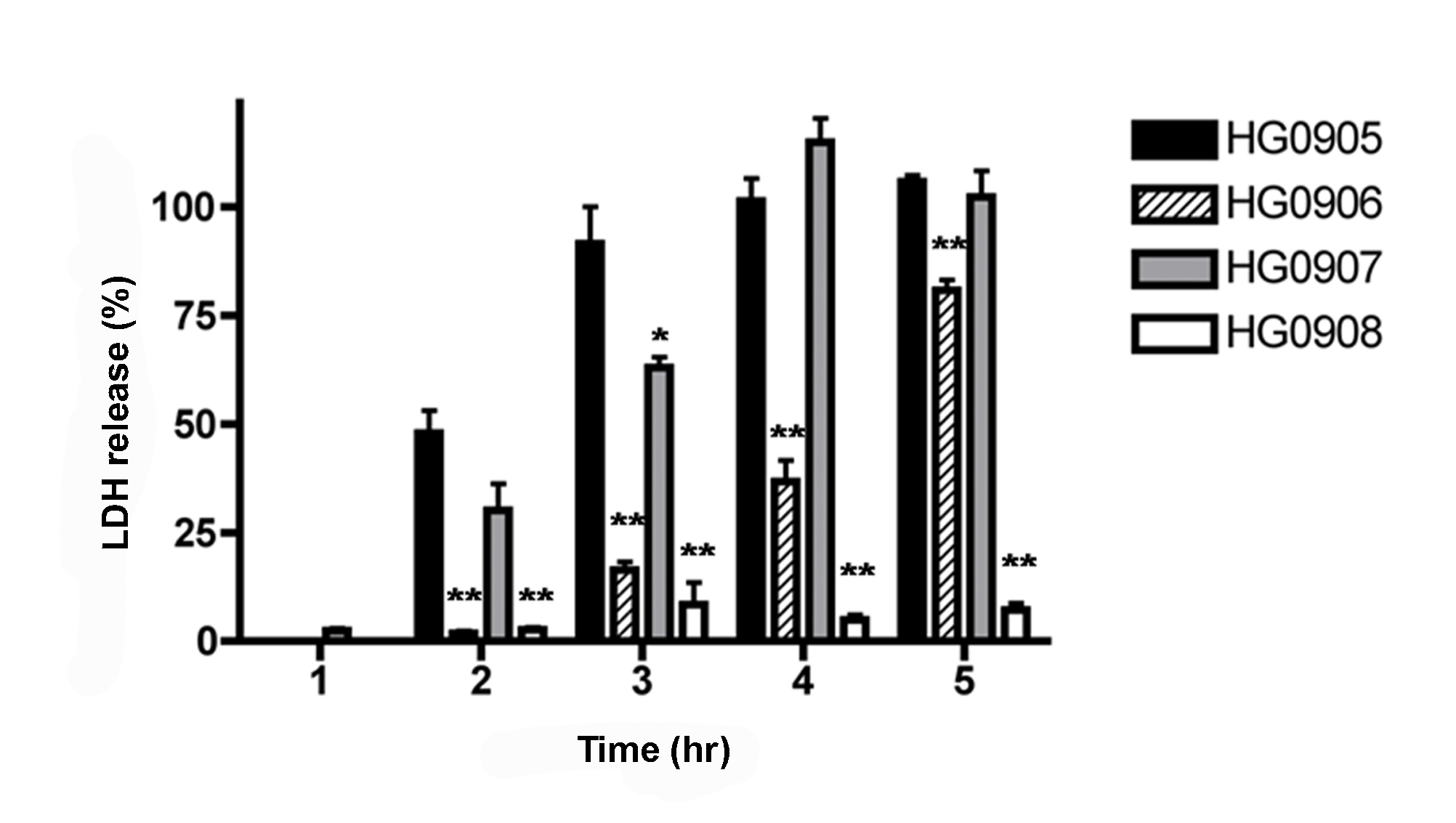

Supplement: Figure S2 — Effect of MARTXVv and VvhA in lux + V. vulnificus on HeLa cell lysis activity. HeLa cells were infected with the HG0905, HG0906, HG0907 and HG0908 at MOI of 25 and LDH activity were determined at various incubation times (*, p<0.05; **, p<0.01). (TIF) [file ppat.1002581.s002.tif]
