# Supplementary material for: Additive Function of Vibrio vulnificus MARTXVv and VvhA Cytolysins Promotes Rapid Growth and Epithelial Tissue Necrosis During Intestinal Infection
Source: PLoS Pathog. 2012 Mar 22;8(3):e1002581. doi: 10.1371/journal.ppat.1002581 (PMC3310748; doi:10.1371/journal.ppat.1002581)
Supplement: Table S1 — Lethality of luciferase-expressing V.vulnificus strains to mice. (DOCX) [file ppat.1002581.s003.docx]

**Table S1. Lethality of luciferase-expressing *V.vulnificus* strains to mice.**

| **Strains^a^** | **LD_50_ (CFU)** |
| --- | --- |
| HG0905 (*n*=10) | 3.1 X 10^5^ |
| HG0906 (*n*=10) | 8.0 X 10^7^ |
| HG0907 (*n*=10) | 1.9 X 10^7^ |
| HG0908 (*n*=10) | > 10^9^ |

^a^ n, number of mice for each inoculation group. Inoculations are ranged from 10^5^-10^9^ CFU in 10-fold increments.
